# The data substrate of exposome intelligence: an interoperability profile for untargeted metabolomics

**DOI:** 10.3389/frai.2026.1901969

**Published:** 2026-08-24

**Authors:** Thomas Hartung

**Affiliations:** 1 Doerenkamp-Zbinden Chair for Evidence-based Toxicology, Bloomberg School of Public Health and Whiting School of Engineering, Johns Hopkins University, Center for Alternatives to Animal Testing (CAAT), Baltimore, MD, United States; 2 CAAT Europe, University of Konstanz, Konstanz, Germany

**Keywords:** data interoperability, exposome, FAIR data, federated learning, high-resolution mass spectrometry, meta-analysis, metabolite annotation, untargeted metabolomics

## Abstract

Untargeted metabolomics, anchored in high-resolution mass spectrometry, has matured into the central analytical platform of human exposomics. It can capture endogenous biology, diet, drugs, microbial chemistry, environmental contaminants, and their transformation products from a single biological sample. Yet exposome science remains stubbornly single-study: most untargeted exposomics publications stand alone, featuring tables, partial annotations, and semi-quantitative intensities that cannot be combined across cohorts. The bottleneck is no longer instrumentation or annotation; it is interoperability. Existing standards, including MSI, mQACC, BP4NTA, NORMAN, MERIT, mzML, mzTab-M, ISA-Tab, the Universal Spectrum Identifier, RefMet, ChEBI, MetaboLights, Metabolomics Workbench, GNPS/MassIVE, and the emerging GA4GH human exposome data standards, cover the necessary ingredients but do not yet compose a single, executable profile. I argue that the next stage of exposomics must move from FAIR deposition to meta-analysis-ready evidence: a four-layer stack of acquisition comparability, machine-readable reporting, evidence-aware annotation, and standardized summary statistics, validated by a living community benchmark. Cumulative exposome science depends on it.

## 1 Introduction: the promise and the bottleneck

**Figure 1:**
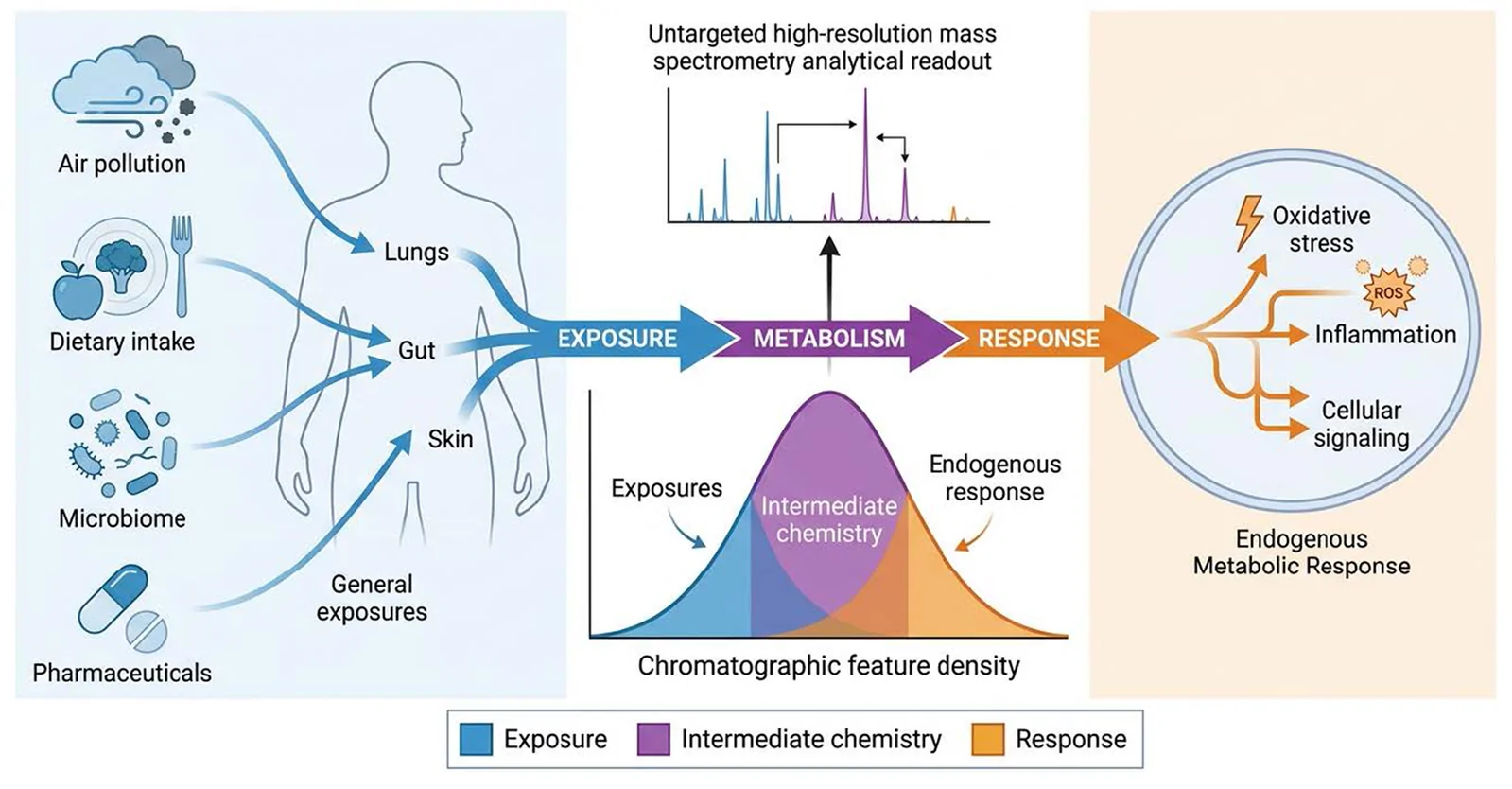
The endo-exo bridge: metabolomics between exposure and phenotype. The metabolome occupies a unique position in human biology, sitting at the intersection of three classes of chemical signals. Exposures and their primary transformation products (*in vivo* and microbial) enter from the left. Endogenous biological responses (perturbed metabolic pathways, oxidative stress, inflammation) enter from the right. Intermediate chemistry connecting the two (drug metabolism, conjugates, secondary transformations) sits in between. Untargeted high-resolution mass spectrometry is the only analytical platform that captures all three classes simultaneously in a single biological sample, which is why metabolomics has emerged as the central platform of human exposomics.

“*The whole is more than the sum of its parts*.” Aristotle (384–322 BC) stated this in *Metaphysics*. We have spent almost two decades arguing that toxicology must escape the confines of single, hand-crafted tests applied to individual chemicals (Hartung, 2009b; Hartung et al., 2013a; Tollefsen et al., 2014; Rovida et al., 2015; Caloni et al., 2022). This aligns with a transition from animal-based, slow methods to big data analyzed with AI and human-relevant testing methodologies to address remaining testing needs. The exposome is the natural culmination of that argument (Sillé et al., 2020; Hartung, 2023). If the genome explains only a minor fraction of disease risk (Rappaport, 2016) and the rest is, in the brutal aphorism, “*bad luck and exposure*,” then a research enterprise that measures one chemical at a time, in one rat at a time, against one health outcome at a time, is inherently unable to deliver the answers public health requires. The exposome promises a different approach. It suggests relaxing the rigor of any single measurement and recovering power through breadth and integration. This promise of the exposome as a new approach to risk assessment (Sillé et al., 2020) calls for a Human Exposome Project (Hartung, 2023). This third article is the methodological consequence of the first two. It is one thing to commit, in principle, to a comprehensive measurement of human exposures and their biological imprints (Figure 1). It is another to make that measurement cumulative, ensuring that the next study can build on the findings of the last.

Untargeted metabolomics has emerged as the central analytical platform for this ambition. High-resolution mass spectrometry, coupled with liquid or gas chromatography, ion mobility, and data-independent acquisition strategies, can now detect tens of thousands of molecular features in a milliliter of plasma or urine. These features include drugs, dietary compounds, metabolites of the gut microbiome, environmental contaminants, and their in-body transformation products, alongside the endogenous biochemistry that responds to all of them (Vermeulen et al., 2020). Mass spectrometry is the only platform we have that can plausibly answer the question that has haunted environmental health for a generation: what is the totality of small molecules circulating in this person today, and what does it say about their exposure history and biological state? Christopher Wild's original framing of the exposome as everything that is not the genome (Wild, 2005) has, in the intervening 20 years, been operationalized largely through metabolomics, beginning with Rappaport and Smith's 2010 *Science* commentary that focused attention on the blood exposome (Rappaport and Smith, 2010; Miller, 2013; Rappaport et al., 2014) and continuing through Miller and Jones's reframing of the exposome as the integral of all environmental forces acting upon our genome (Miller and Jones, 2014).

The good news is that the technology has, by any reasonable standard, arrived. The Vermeulen *Science* review put it succinctly when it titled its central thesis “*where chemistry meets biology*” (Vermeulen et al., 2020). We can routinely acquire high-resolution tandem mass spectra at scale, run them through public networking platforms such as GNPS^1^ (a consolidated list of all tools, standards, and resource URLs is provided in Supplementary Appendix A). (Wang et al., 2016) or open-source structural annotation engines such as SIRIUS^2^ (Dührkop et al., 2019, 2021), interrogate continually growing spectral libraries, and deposit raw spectra in repositories that now exceed eight thousand metabolomics studies (Yurekten et al., 2024). High-resolution non-target screening in environmental samples is a discipline in its own right, with formal guidance documents from the NORMAN network^3^ (Hollender et al., 2023). For the first time, a single biological sample can be reasonably claimed to contain a discovery-grade representation of the chemical exposome.

Before turning to that bottleneck, it is worth acknowledging that the analytical maturity just described coexists with well-recognized and still-unresolved general limitations of untargeted metabolomics as a technology. Metabolite annotation remains incomplete and uncertain, with only a minority of detected features confidently identified in a typical study. Quantitative accuracy is constrained by matrix effects, variable ionization efficiency, and the scarcity of authentic standards. Reproducibility across instruments, laboratories, and vendor platforms is imperfect, and cross-study comparability is limited by all of the above. A recent comprehensive review of functional metabolite annotation catalogs these challenges in detail and underscores that annotation in particular remains a rate-limiting step for the field (Nguyen et al., 2024). These general limitations provide the backdrop against which the more specific interoperability problem addressed in this article should be read: even where the underlying measurement is excellent, the outputs of different studies remain difficult to combine.

The bad news is that we cannot yet routinely combine these data. It is important to emphasize the distinction between the discovery state of the art, which is impressive, and the synthesis state of the art, which is weak. Almost every untargeted exposomics study is, in practice, an island. Its feature table partially overlaps with feature tables in other studies. Its annotations range between confirmed structures and class-level guesses, with the confidence calls described in the methods but lost in downstream summary tables. Its intensities are relative, not absolute, and cannot be compared in magnitude across instruments. Its quality control regime varies by laboratory. Its metadata are recorded in the journal's tradition of narrative methods rather than in a machine-readable form. Its raw data may or may not be deposited, and even when deposited may not be reusable because file formats, vendor configurations, batch structure, or sample metadata are inadequate. Two studies that ostensibly measure “*untargeted metabolomics in plasma*” can be, for the purpose of meta-analysis, as different as cuneiform and emoji.

The field today can be summarized in a single sentence: untargeted exposomics has reached the point where the limiting factor is no longer analytical discovery but evidence integration. That is the central argument of this article. The next stage of exposomics must move from “*single-study discovery*” toward FAIR, federated, meta-analyzable exposome science. The field has the ingredients. It does not yet have the recipe.

By “recipe,” I mean something quite specific. I do not mean another checklist. The exposomics community already has more checklists than it can implement (MSI Board Members et al., 2007; Goodacre et al., 2007). I mean an executable interoperability profile: a small set of machine-readable formats, controlled vocabularies, validators, repository requirements, and statistical export schemas that, in combination, permit cross-study meta-analysis without re-acquiring the data. This is both the challenge and the opportunity for the Global Exposome Forum,^4^ which developed out of our Exposome Moonshot Forum^5^ (Sillé and Hartung, 2025; Anderer, 2025; Stetler, 2025). I will use the rest of this article to advocate for that profile, map it against the existing standards, and propose a community demonstration agenda that would determine whether the profile is fit for purpose. The article is structured into 12 numbered sections. Sections 2 and 3 explain why metabolomics is the right backbone for the exposome and assess the current state of the art. Sections 4 and 5 outline the existing standards landscape and diagnose why current studies cannot be combined. Sections 6 and 7 argue for two conceptual shifts: from feature names to evidence objects, and from intensity tables to a five-tier quantification scheme. Section 8 catalogs the five forms of meta-analysis that are feasible today and their trade-offs. Section 9 presents the central conceptual contribution of this piece: the exposome–metabolome evidence stack. Section 10 lays out a reporting standard for meta-analysis-ready exposomics. Section 11 proposes a community-scale demonstration, the Exposomics Meta-Analysis Living Benchmark. Section 12 connects the proposal to regulatory toxicology, public health practice, and the Human Exposome Project, offering concrete steps an individual laboratory can take this year. A short Conclusions section closes the argument.

It is also worth noting what this article is not. It is not a systematic review of metabolomics methods in exposomics; the field has those, and recent reviews are excellent (Vermeulen et al., 2020). It is not a technical specification; specifications are properly produced by community working groups, not by single authors, and the existing groups [mQACC,^6^ BP4NTA,^7^ MERIT,^8^ NORMAN (see text footnote 3), HUPO-PSI,^9^ GA4GH^10^] are far better positioned to draft the eventual profile than I am. What I hope this article will do is identify the missing piece, outline its shape, and provide the political and scientific motivation for the working groups to assemble it.

## 2 Why metabolomics is the right backbone for exposomics

The exposome was originally conceived as a measurement program, not as an organ system. Wild's 2005 paper proposed exposomics as a counterpart to genomics: where genomics catalogs heritable material, exposomics should catalog everything else that determines the biology of an individual organism (Wild, 2005). That definition is appropriate as a target but unhelpful as a measurement strategy because “*everything else*” is a moving target with no obvious instrumentation. Rappaport's reformulation of the exposome as the totality of chemicals measurable in blood (Rappaport and Smith, 2010; Rappaport et al., 2014) was the first decisive step toward operationalization, as it tied the abstract concept to an actual analytical platform. The platform, increasingly, is high-resolution mass spectrometry, and the language is metabolomics (Sillé and Hartung, 2024). Two conceptual articles have addressed how key demands for the further development of metabolomics to serve the Human Exposome can be met: the identification of more features and their semi-quantification (Sillé et al., 2025, 2026a,b).

Why metabolomics rather than, say, proteomics or genomics-as-exposure-fingerprint? The answer comes from a simple thought experiment. Pick any of the hundreds of millions of chemical entities in modern chemical registries. Ask which omics platform is most likely to detect it in a human biofluid. For exogenous small molecules and most of their primary in-body transformation products, the answer is mass spectrometry-based metabolomics, almost by definition: these molecules are small, polar or moderately lipophilic, ionizable in electrospray or chemical ionization, and present at concentrations within the dynamic range of modern triple-quadrupole and high-resolution instruments. Proteomics will detect adducts and modifications to plasma proteins, but it will not see the parent compound. Genomics and epigenomics will detect inherited risk and chromatin marks, but they are blind to the chemical species responsible for those marks. Transcriptomics will detect biological responses to exposure, but again will not see what hit the cell. Metabolomics is the only platform that can, in a single assay, capture the exogenous chemical, its biological transformation, and at least some of the endogenous response. That is its disciplinary privilege.

The privilege comes with an interpretive problem that the field has spent two decades wrestling with. A peak in an untargeted metabolomics chromatogram does not, by itself, indicate whether it is an exposure, a response, an intermediate, or noise. The same plasma metabolite may originate from environmental contamination, a co-administered drug, the gut microbiome's metabolism of dietary precursors, or an endogenous pathway perturbed by a stressor. Vermeulen et al. described this directly when they characterized the exposome–metabolome as the intersection of chemistry and biology (Vermeulen et al., 2020). The dual-origin problem is not a flaw of the metabolomics approach; it is the actual structure of human exposure. The challenge for the field is to design analyses that can extract exposure information from a signal with an uncertain origin while also extracting response information from the same signal. I will return to this in Section 6, but the basic point belongs here: metabolomics is the natural backbone of exposomics precisely because it does not segregate exposure from biology. They are entangled in the data because they are entangled in life.

There is a second reason for centering metabolomics in exposomics, which is practical. The cost per sample for untargeted high-resolution LC-MS or GC-MS analysis is now well below the cost of comprehensive targeted panels for the same chemical breadth. NORMAN's recent guidance (see text footnote 3) on non-target screening pointed out that routine targeted environmental monitoring covers tens to hundreds of analytes, while non-target screening in the same sample can detect thousands of features, a substantial fraction of which can be tentatively assigned to at least a chemical class (Hollender et al., 2023). Within the human population, the chemical exposure space is plausibly larger, not smaller, than the environmental exposure space because human bodies host their own metabolism and microbial flora that further transform every input. The targeted approach cannot keep up. If we want a measurement program that scales to the actual chemical complexity of human exposure, untargeted high-resolution mass spectrometry is the only candidate that operates at the necessary throughput.

Care should be taken not to overclaim. Untargeted metabolomics is not a substitute for the rest of exposomics. Air pollution monitoring, wearable sensors, residential exposure indices, dietary recall instruments, occupational hygiene records, and social determinant data layers are all part of the exposome (Sillé et al., 2020; GA4GH Human Exposome Data Standards Study Group, 2024). These external measurements anchor and explain internal signals. Without them, metabolomic features are uninterpretable. Without metabolomic features, external measurements cannot detect mixtures, transformation products, and the many exposures we forget to instrument. The two streams are complementary, and the GA4GH human exposome data standards effort has rightly emphasized the need to develop interoperable schemas across both external and internal exposures, linked to phenotypic data through the existing Phenopackets standard (GA4GH Human Exposome Data Standards Study Group, 2024). The argument for centering metabolomics in this article is not that other streams do not matter. The argument is that metabolomics generates the kind of high-dimensional, internally validated, chemistry-rich data layer for which interoperability is hardest, and therefore most urgent.

The conceptual upshot of this section is short. Metabolomics is the endo-exo bridge of exposome science (Figure 1). It sees exposures, it sees responses, and it sees the intermediate chemistry that connects them. It is the only analytical platform that can occupy this bridge at the necessary throughput and chemical breadth. For exactly that reason, it is also the place where interoperability gaps cost the most, because failing to standardize a high-dimensional data layer that bridges exposure and biology is failing to standardize the very heart of the exposome.

## 3 Where the state of the art actually is

Before discussing what needs to change, I want to be honest about what does not. The technical state of the art in untargeted metabolomics-based exposomics is, by any defensible measure, strong. I want to enumerate what is working before turning to what is missing, because too many calls to action in this field begin by underselling the foundation they are about to recommend building on. The foundation is real.

On the acquisition side, modern high-resolution mass spectrometers routinely deliver sub-ppm mass accuracy in both positive and negative electrospray ionization modes, with MS/MS spectra acquired in data-dependent or, increasingly, data-independent modes, and run-to-run reproducibility that, with appropriate quality controls, supports cohort-level analyses of hundreds to thousands of samples. Ion mobility separation, layered on top of chromatography, adds an orthogonal collision cross-section dimension that helps distinguish isomers and improves library matching (Picache et al., 2019). Time-of-flight and Orbitrap geometries now reach resolutions that allow confident assignment of molecular formulas for the great majority of detected ions below mass-to-charge 500. The combination of soft ionization with high-resolution tandem mass spectrometry has made the routine detection of low-abundance xenobiotics in complex biofluids a solved problem at the analytical level, even if it remains an unsolved problem at the synthesis level.

On the annotation side, the picture is similarly encouraging. Schymanski et al.' five-level confidence scheme for high-resolution mass spectrometry identification has been adopted across the non-target chemistry and metabolomics communities and provides the *lingua fran*c*a* for discussing how confident an assignment actually is (Schymanski et al., 2014). The Global Natural Products Social Molecular Networking platform [GNPS/MassIVE (see text footnote 1)] connects spectral library search, molecular networking, and community-curated annotation in a single ecosystem that now hosts more than a billion tandem spectra across thousands of public datasets (Wang et al., 2016; Nothias et al., 2020; Schmid et al., 2021). The SIRIUS framework (see text footnote 2) integrates formula prediction with structural inference through CSI:FingerID and with class-level inference through CANOPUS,^11^ allowing meaningful annotation even when an exact spectral library match is unavailable (Dührkop et al., 2019, 2021). MSNovelist^12^ (Stravs et al., 2022) and related tools now attempt structural annotation for compounds missing from spectral libraries entirely by generating candidate structures from fragmentation patterns (Hoffmann et al., 2022). For environmental contaminants specifically, the NORMAN Suspect List Exchange^13^ aggregates tens of thousands of substances into harmonized lists that can be searched as suspects in non-target screens (Taha et al., 2022). The combined effect is that, for any feature in a modern untargeted dataset, there is some probability of obtaining at least a class-level annotation, and a smaller but real probability of obtaining a confirmed identification.

On the data side, repositories now exist for both raw and processed data. MetaboLights,^14^ hosted at EMBL-EBI, requires raw experimental data and ISA-structured metadata for every study and has grown from 1,432 studies in early 2020 to more than eight thousand by late 2023 (Yurekten et al., 2024). The NIH-funded Metabolomics Workbench^15^ requires similar deposition, using its own mwTab format, and provides curated study metadata across thousands of human and model-organism studies (Sud et al., 2016). GNPS/MassIVE (see text footnote 1) hosts a complementary stream of MS/MS data with community-driven re-analysis infrastructure (Wang et al., 2016). The proteomics standards initiative, HUPO-PSI, has produced the central open formats for mass spectrometry raw data (mzML) and for processed results (mzTab-M), with mzTab-M version 2.0 specifically designed to capture quantitative metabolomics results together with the evidence trail for identifications, including ambiguity and adduct hypotheses (Hoffmann et al., 2019). The Universal Spectrum Identifier was ratified by HUPO-PSI in 2021 and provides a globally resolvable way to cite an individual mass spectrum across repositories (Deutsch et al., 2021). Recent work has begun to enable genuine pan-repository reanalysis across MetaboLights, Metabolomics Workbench, and GNPS/MassIVE (see text footnote 1) demonstrating that the infrastructure for cross-repository discovery has finally caught up with the volume of deposited data (Hosseini et al., 2025).

On the quality side, the field has been moving toward consensus for a decade. The first dedicated effort to articulate quality-assurance requirements for metabolomics in the context of toxicology was the t^4^ workshop hosted at the Johns Hopkins Center for Alternatives to Animal Testing in November 2013, with members of the NIH Human Toxome Consortium and invited experts from academia, industry, and regulatory agencies. The resulting report (Bouhifd et al., 2015) outlined the challenges of quality assurance from experimental design through bioinformatics and explicitly anticipated the need for a coordinated community effort. That effort took institutional form in 2018 with the founding of the Metabolomics Quality Assurance and Quality Control Consortium (mQACC), whose first community report described the development steps and priority objectives of a coordinated QA/QC initiative (Beger et al., 2019). The Best Practices for Non-Targeted Analysis working group (BP4NTA) has produced parallel resources for environmental non-targeted analysis, including the Non-Targeted Analysis Study Reporting Tool, which provides a structured framework for reporting study design and quality assessment (Place et al., 2021; Peter et al., 2021). The Metabolomics Standards Initiative in Toxicology [MERIT (see text footnote 8)] has published best-practice and reporting standards specifically for regulatory toxicology applications of metabolomics, with explicit linkage to the OECD Metabolomics Reporting Framework that is becoming the basis for regulatory acceptance of omics data in chemical safety assessment (Viant et al., 2019; OECD, 2023).

This is, by any measure, a richly populated foundation. The exposomics community has, over 20 years, assembled the building blocks of a cumulative discipline: it has open file formats, public repositories, controlled vocabularies, quality control consortia, confidence schemata, structural annotation engines, and a regulatory acceptance framework. What it does not yet have, in operational form, is an integrated profile that combines these building blocks into something a single laboratory can deploy and a meta-analyst can rely upon. The result is the curious situation in which the field appears, from a distance, to be a mature interoperability ecosystem, while up close it remains a collection of well-built but disconnected islands. The next section maps those islands in more detail and explains where the bridges are missing.

## 4 The standards landscape: many ingredients, no recipe

**Figure 2:**
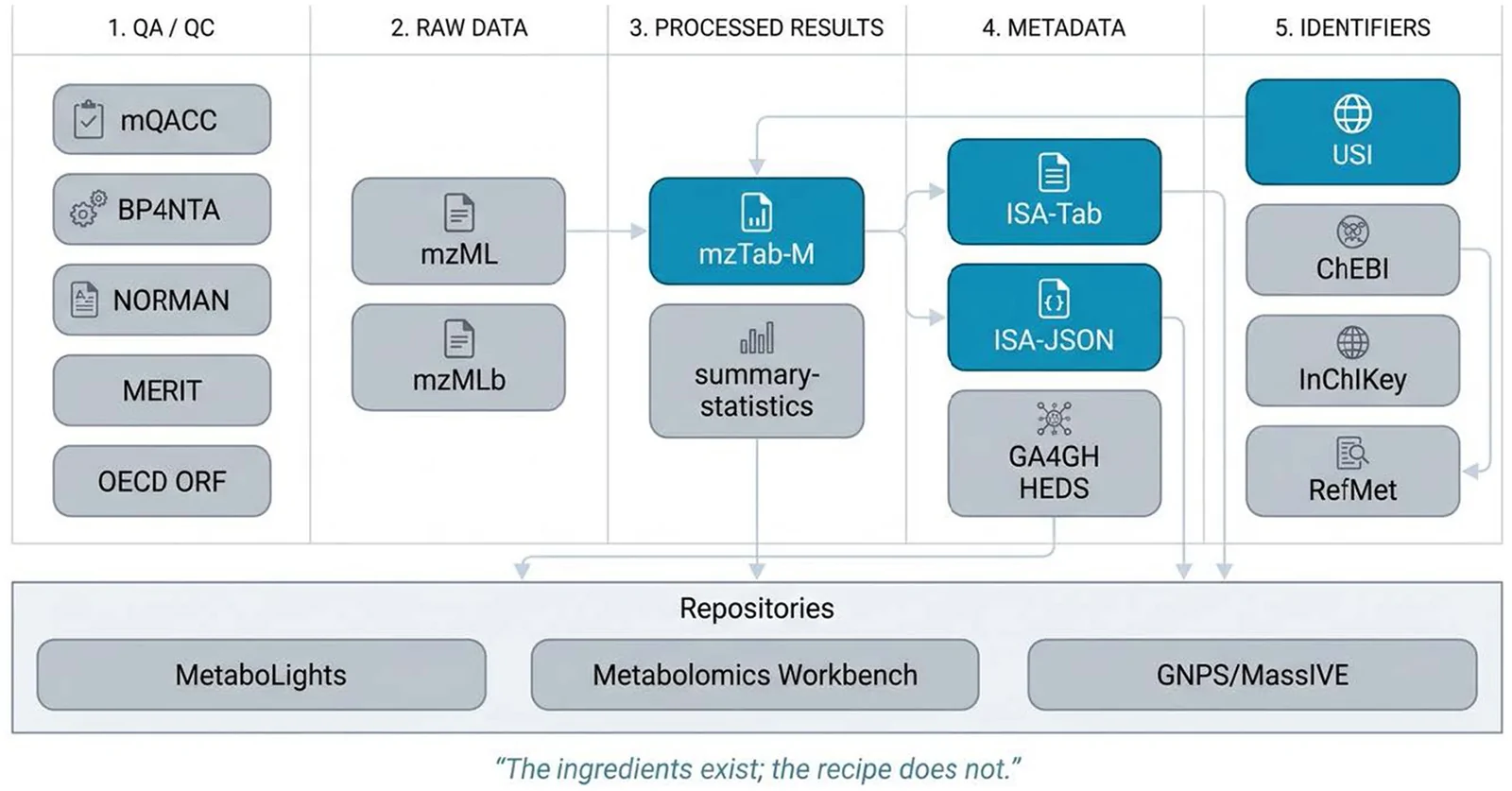
The standards landscape map. The exposomics community has produced a rich but fragmented landscape of standards, organized here by their coverage areas. Five vertical columns represent the major coverage areas: quality assurance and quality control **(Left)**, raw data formats, processed-result formats and identifiers, study and assay metadata, and chemical and spectral identifiers **(Right)**. Each existing standard or framework is shown as a labeled rectangle within the appropriate column, with arrows indicating where standards reference or extend each other. The figure illustrates the central diagnostic of this article: the ingredients exist; the recipe does not.

A common mistake in standards advocacy is to write as if the field had no standards. The exposomics community has many (Figure 2). The problem is not their absence; it is fragmentation. The standards exist in different documents, produced by different working groups, hosted on different web pages, with varying scopes, vocabularies, validators, and adoption levels. A laboratory that wishes to do “*everything right*” must read perhaps a dozen separate documents and then guess how to harmonize them. A meta-analyst who wishes to pool data across cohorts must reverse-engineer this harmonization for studies that, in most cases, did not perform it. This section walks through the major standards and points out where they speak past each other.

Begin with the foundational text. The original Metabolomics Standards Initiative (MSI^16^), led by Sansone, Fan, Goodacre, and collaborators in 2007, produced a set of minimum reporting recommendations for metabolomics studies, covering study design, sample handling, chemical analysis, and data processing (MSI Board Members et al., 2007; Goodacre et al., 2007). MSI level definitions for metabolite identification, in particular, became widely cited in the metabolomics community. They predate the era of routine high-resolution mass spectrometry and were extended, particularly for non-target HRMS, by Schymanski et al. (2014) five-level confidence scheme. Both frameworks remain in use; they overlap but are not identical, and many studies cite one without acknowledging the other, which complicates cross-study comparisons of “*level 2*” identifications.

Next, the quality and reporting standards. The first dedicated workshop articulating quality-assurance requirements for metabolomics in toxicology took place in 2013 (Bouhifd et al., 2015); its goals were to consider the challenges of metabolomics as an emerging analytical science and to identify the key issues to be addressed in establishing QA procedures for metabolomics-based toxicology. mQACC, founded in 2018 with several of the same investigators, has since focused on quality assurance and quality control practices for untargeted metabolic phenotyping at the consortium scale (Beger et al., 2019). Its outputs include an analysis of QA/QC practices among LC-MS-based untargeted metabolomics laboratories, surveying maintenance, calibration, data archival, repository deposition, standard operating procedures, system suitability procedures, pooled-QC use, internal standard use, and other practices (Evans et al., 2020); a review of reference materials for the field covering matrix-based and synthetic standards, NIST reference materials, and lipidomics-specific materials (Lippa et al., 2022); and recommendations for analytical quality management addressing instrument suitability, system equilibration, batch design, blank handling, signal correction, and reference material selection. BP4NTA, founded contemporaneously for environmental non-target analysis, has produced reporting recommendations and a study reporting tool that overlap substantially with mQACC's guidance but use different vocabulary and emphasize environmental matrices and suspect lists (Place et al., 2021; Peter et al., 2021). NORMAN's guidance on suspect and non-target screening covers similar ground from the environmental monitoring perspective and explicitly addresses the use of suspect lists, retention time indexing, and confidence reporting (Hollender et al., 2023). MERIT (see text footnote 8), anchored at Oxford and contributing to OECD frameworks, focuses on the regulatory toxicology use case and emphasizes the derivation of points of departure, mechanism of action, read-across, and cross-species extrapolation (Viant et al., 2019). The OECD Omics Reporting Framework synthesizes elements from these efforts into a regulatory-acceptable format for transcriptomics and metabolomics, although uptake in actual regulatory submissions remains limited (OECD, 2023).

For data formats, the central layer is provided by HUPO-PSI. mzML is the open community standard for mass spectrometry raw data (Martens et al., 2011), with mzMLb providing an HDF5-based variant that supports random access and is increasingly attractive for very large datasets (Bhamber et al., 2021). mzTab-M version 2.0 is the standard for reporting processed metabolomics results, including quantitative values, evidence trails, controlled vocabulary references, and explicit ambiguity in identifications (Hoffmann et al., 2019). The Investigation-Study-Assay framework (ISA-Tab^17^ and ISA-JSON) provides a hierarchical metadata schema that MetaboLights uses for study curation and is broadly applicable across omics types (Sansone et al., 2012). The Universal Spectrum Identifier, ratified by HUPO-PSI in 2021, provides a globally resolvable spectral identifier that allows any specific mass spectrum in any participating public repository to be cited (Deutsch et al., 2021). For chemical identity, ChEBI provides an open ontology of chemical entities with names, formulas, InChI, SMILES, and cross-references (Hastings et al., 2016); InChIKey hashes provide stable identifiers across databases (Heller et al., 2015); RefMet^18^ provides a normalized nomenclature for metabolite names that addresses the chronic problem of inconsistent naming across studies (Fahy and Subramaniam, 2020).

For repositories, MetaboLights and Metabolomics Workbench cover the bulk of human and model-organism metabolomics deposition, while GNPS/MassIVE (see text footnote 1) focuses on tandem mass spectral data and re-analysis (Yurekten et al., 2024; Sud et al., 2016; Wang et al., 2016). The recent Pan-ReDU effort has begun to link these three repositories under harmonized metadata for re-analysis (Hosseini et al., 2025). The Metabolomics Workbench file status website has also made a quiet but important advancement by providing validation logs that flag inconsistencies in deposited files and allow meta-analysts to filter studies based on adequate file integrity (Powell and Moseley, 2023).

For exposure-specific data layers, the situation is still developing. The GA4GH Human Exposome Data Standards Study Group, launched within the Global Alliance for Genomics and Health, is the first serious attempt to define interoperable schemas for external exposures (air pollution indices, satellite-derived environmental measurements, wearables-derived metrics, social determinants) and internal exposures (metabolomics, microbiome, diet, drug intake) in a way that connects to the existing Phenopackets standard for phenotypes (GA4GH Human Exposome Data Standards Study Group, 2024). The European Human Exposome Network (a cluster of nine projects funded under Horizon 2020 and continued under Horizon Europe) has produced complementary tools and protocols, including ATHLETE,^19^ EXIMIOUS,^20^ EPHOR,^21^ EQUAL-LIFE,^22^ EXPANSE,^23^ HEAP,^24^ HEDIMED,^25^ LongITools,^26^ and REMEDIA^27^ (Vlaanderen et al., 2021). The Human Biomonitoring for Europe initiative (HBM4EU^28^) and its successor PARC^29^ (Marx-Stoelting et al., 2023) have produced harmonized protocols for targeted analyte panels that complement untargeted approaches (Ganzleben et al., 2017).

I have enumerated roughly 15 distinct standards efforts across five overlapping domains, none of which subsume the others, and many of which do not explicitly cross-reference one another. The result is a landscape that appears as a successful federation from the perspective of any individual working group but resembles a labyrinth for anyone trying to navigate it. In the author's view, this is the central practical problem the field needs to address. The proposed solution is not another working group producing another checklist; rather, it is a crosswalk: an explicit mapping among the existing standards that indicates, for any given reporting requirement, which standard covers it, which terms are normative, where the validators are located, and how the outputs contribute to a meta-analysis-ready summary layer.

I will outline such a crosswalk in Section 9, after first explaining, in Sections 5 through 8, why the absence of such a crosswalk is not a cosmetic inconvenience but the active reason untargeted exposomics studies cannot currently be combined.

## 5 Why current untargeted exposomics studies do not combine

**Figure 3:**
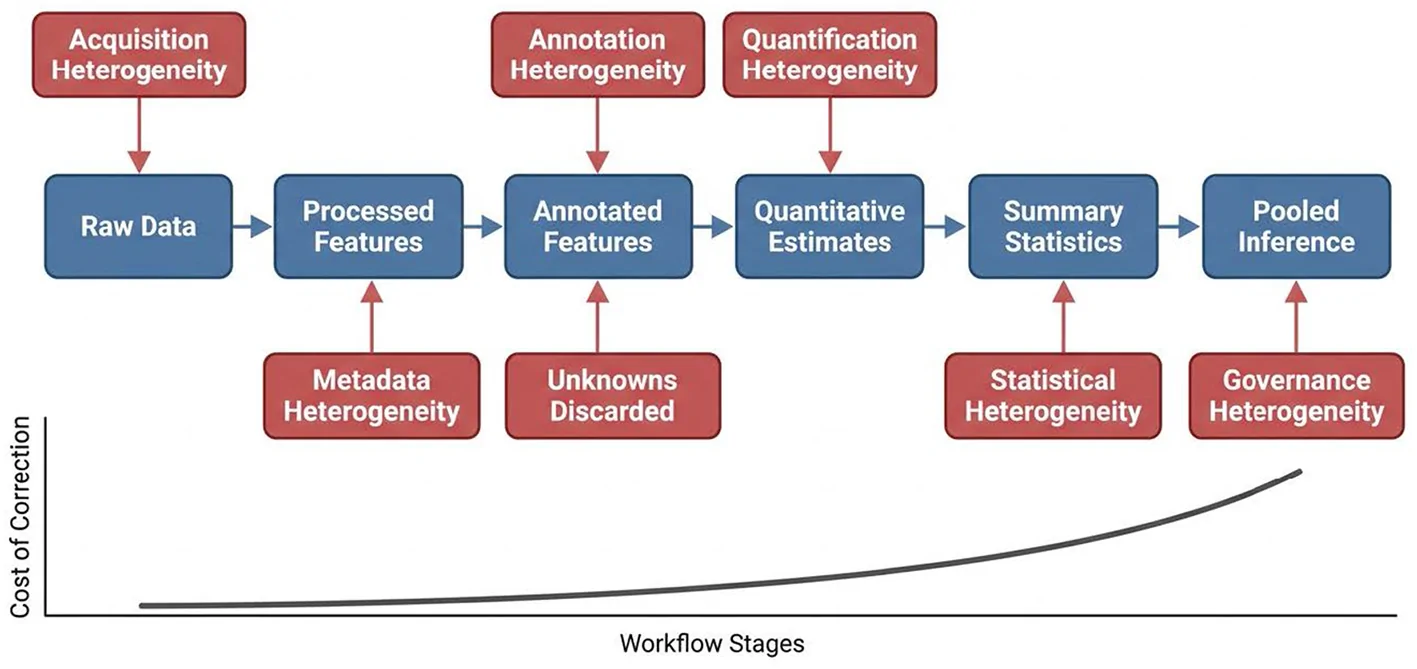
Why current studies do not combine: the seven failure modes. Seven failure modes obstruct the cross-study combination of untargeted exposomics data. Each enters the meta-analytic pipeline at a characteristic point: acquisition heterogeneity at the start and governance heterogeneity at the end. The figure traces a generic meta-analytic workflow (left to right: raw data → processed features → annotated features → quantitative estimates → summary statistics → pooled inference) and indicates where each failure mode interrupts the flow. The downstream cost of each failure mode increases as the workflow proceeds, because errors at later stages are harder to remedy without re-acquiring data.

If standards exist, why are studies not combinable? The answer lies in the gap between “*standards published*” and “*standards practiced*,” and in the further gap between “*standards practiced*” and “*outputs combinable*.” I want to walk through the specific failure modes (Figure 3), because the diagnosis determines the prescription.

The first failure mode is acquisition heterogeneity. Two studies labeled “*untargeted metabolomics in plasma*” can differ on every variable that matters. Chromatography may be reversed-phase C18, HILIC, mixed-mode, or some laboratory-developed variant. Ionization may be electrospray positive only, electrospray negative only, both, or APCI. The mass analyzer may be Orbitrap, time-of-flight, quadrupole-Orbitrap, ion-trap, or a quadrupole time-of-flight hybrid. Acquisition may be data-dependent or data-independent, with the latter implemented as SWATH, MSE, all-ions, or a vendor-specific variant. Collision energies, isolation windows, scan ranges, polarity-switching frequencies, and lock-mass strategies vary. None of these choices is wrong; each is reasonable for some scientific question. However, the practical consequence is that the feature space of one study is, in general, neither a subset nor a superset of the feature space of another. Many features detected in one study do not appear in another simply because the acquisition was not configured to see them.

The second failure mode is annotation heterogeneity. Even when two studies measure overlapping features, their annotations diverge. One may report only confirmed identifications at MSI level 1 or Schymanski level 1, while another may report putative identifications at MSI level 2 or 3, with confidence calls that are not visible in the supplementary tables. A third may rely on class-level annotations from CANOPUS or molecular networking with no reference to MSI levels at all. The same chemical entity may appear as “*L-carnitine*,” “*carnitine*,” “*(R)-carnitine*,” “*Carnitine [M*+*H]*+,” or by a database identifier in a vocabulary the meta-analyst has never seen. RefMet was created to address this kind of nomenclatural drift (Fahy and Subramaniam, 2020), but its adoption is partial; supplementary tables continue to present metabolite names in whatever form the laboratory's processing software emitted.

The third failure mode is quantification heterogeneity. Most untargeted metabolomics intensities are semi-quantitative at best. They are integrated peak areas, normalized by some combination of internal standards, total ion current, probabilistic quotient normalization, or pooled-QC-based correction. Two studies can produce very different numerical intensities for the same chemical entity at the same biological concentration. Absolute quantification is the exception, achieved only when authentic standards and matched-matrix calibration curves are applied, and most untargeted exposomics studies do not perform this for the majority of features. NORMAN's guidance has acknowledged this directly, noting that quantitative non-target analysis is affected by response factors, peak integration definitions, calibration range, matrix effects, and instrument conditions, and that quantification without reference standards is a recognized challenge (Hollender et al., 2023). The downstream consequence is that effect sizes reported as “*log fold-changes in relative intensity*” cannot be straightforwardly pooled across studies that ionized, normalized, or integrated differently.

The fourth failure mode is metadata heterogeneity. Even when raw data are deposited, the metadata necessary for their use are often insufficient. Sample provenance, collection time, fasting status, storage history, blank scheme, batch structure, pooled-QC injection scheme, and blank subtraction protocol may be incompletely captured. Cohort-level metadata are even more heterogeneous: age, sex, BMI, smoking status, medication, dietary recall, residential exposure, and socioeconomic variables are recorded differently in various studies, using different categorizations, scales, and missing-data conventions. Meta-analysis-ready data require these covariates to be harmonized; in practice, they are typically reported in narrative methods with no controlled vocabulary.

The fifth failure mode is statistical heterogeneity. Two studies analyzing the same exposure–metabolite relationship may use different models, with different covariate adjustments, transformations, handling of missingness, multiple-testing corrections, and effect size definitions. One may report fold-change with a Wilcoxon *p*-value; another may report a beta coefficient from a linear model with sex, age, and BMI adjustment; a third may report a partial least-squares variable importance score with no formal hypothesis test. The numbers are not directly poolable. A summary-statistic meta-analysis requires that each study has produced, at minimum, an effect estimate, a standard error, a model specification, and an enumerated covariate set. Most untargeted exposomics studies have not produced any of these in a structured way.

The sixth failure mode is the unknowns problem. A large fraction of features in any untargeted exposomics dataset are not confidently annotated. Some are putative compounds at MSI level 2 or 3, some are class-level annotations, and some are recurring unknowns that appear consistently in many samples but have no spectral library match. In conventional meta-analysis, unknowns are discarded because there is no consistent way to refer to them across studies. This creates a serious bias. Unknowns include precisely the novel exposures and transformation products that exposomics aims to discover. Discarding them reduces untargeted exposomics to a form of targeted exposomics, limited to whatever has been annotated. The Universal Spectrum Identifier, in principle, allows a recurring unknown to be cited with the same precision as a confirmed compound (Deutsch et al., 2021), but the practice of citing unknowns by USI across studies is essentially non-existent today.

The seventh failure mode is governance heterogeneity. For human cohort studies, raw data may not be shareable at all because of consent restrictions, data protection regulations, biobank governance, or institutional policy. In such cases, meta-analysis must be federated: each cohort holds its data locally and exposes only summary statistics or model outputs to a central analyst. Federated meta-analysis is feasible only when the participating cohorts have harmonized their variables, models, and exports, which again requires the very interoperability profile this article argues for. Shared cloud-hosted environments such as the Human Toxome Collaboratorium (Fasani et al., 2016) have demonstrated that the technical infrastructure for cross-institution analytical collaboration is possible, but they have also shown that the more challenging constraint is the harmonization of variables and exports rather than the provisioning of compute.

The cumulative effect of these seven failure modes (Figure 3) is that the meta-analyst encountering a typical pair of published untargeted exposomics studies faces a problem before any statistical step can begin: the impossibility of even establishing a common variable definition. Most of the actual time spent in cross-study analyses is consumed not in meta-analytic statistics but in upstream harmonization. The Cochrane handbook's classic guidance on combining heterogeneous studies (Higgins et al., 2003) addressed precisely this concern for clinical trials, and the resulting investment in reporting standards (CONSORT, PRISMA) is now considered foundational. Exposome science needs an equivalent investment. The next sections argue for the conceptual and technical shifts that would constitute that investment.

## 6 From feature to evidence object: rethinking annotation

**Figure 4:**
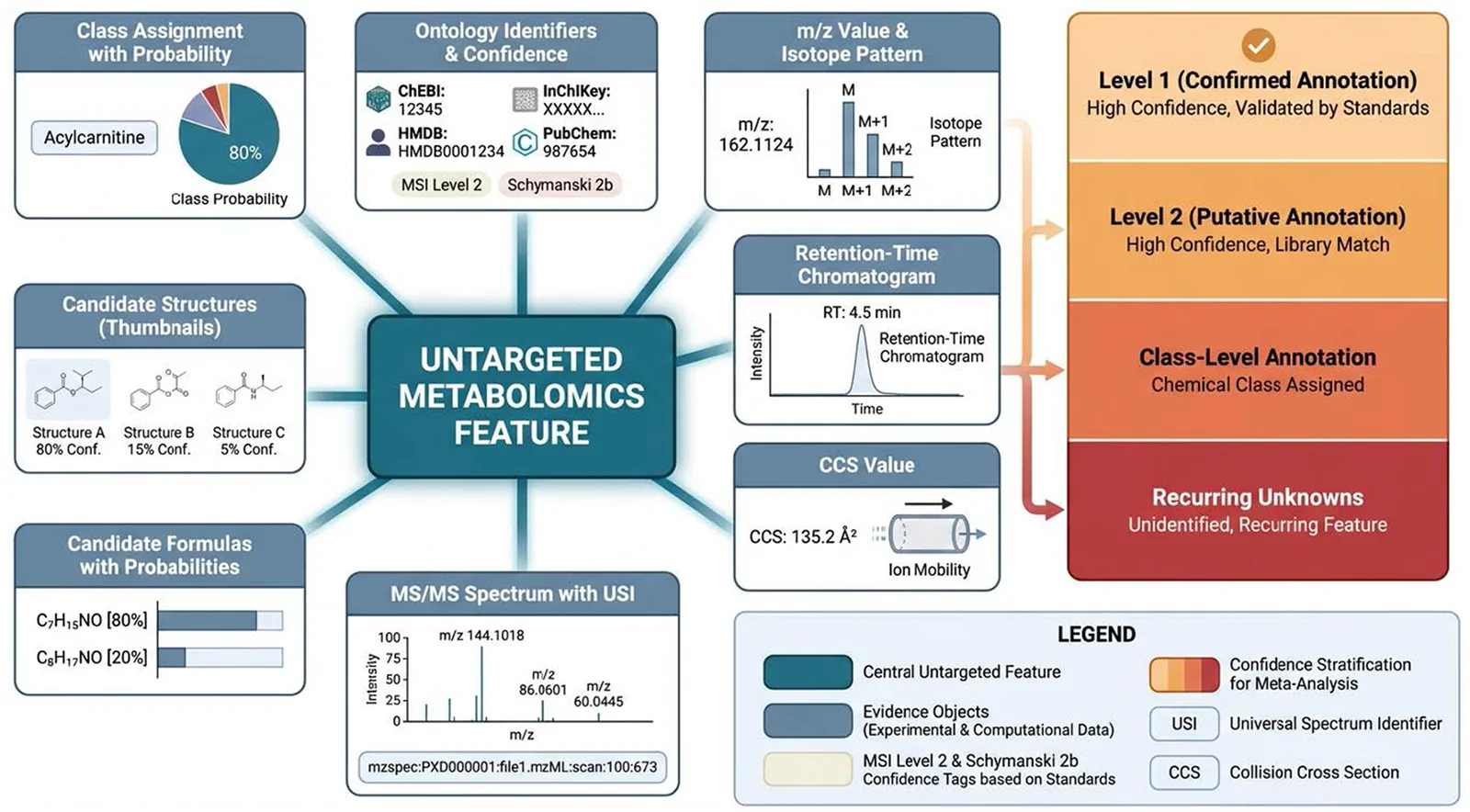
Annotation as evidence object. In conventional practice, an untargeted metabolomics feature is reported as a name (e.g., “carnitine”) and a confidence level (e.g., “MSI Level 2”). This diminishes the information needed for cross-study meta-analysis. The figure shows the alternative: each feature is represented as a structured evidence object carrying all the supporting evidence (m/z, retention time, collision cross-section where available, MS/MS spectrum with USI, formula and structural candidates with probabilities, chemical-class assignment with probability, ontology identifiers, and explicit confidence levels under both MSI and Schymanski schemes). With this representation, downstream meta-analysis can stratify by confidence and integrate features at appropriate levels of certainty.

The most important conceptual move this article urges the field to make is to stop treating an annotation as a name and start treating it as an evidence object (Figure 4). This is not a software question; it is a question of what counts as the unit of analysis.

In current practice, a feature in an untargeted dataset is processed into a row of a results table with a few columns: intensity, putative identification, perhaps a confidence level, and perhaps a pathway assignment. The downstream analyst pools or compares these rows. What is lost in this step is the evidence behind the identification, which precisely determines whether two laboratories agree about the same molecule. The mass-to-charge value, the observed retention time, the adduct hypothesis, the isotopic pattern, the MS/MS spectrum, the library match score, the *in silico* annotation score, the candidate formulas, the candidate structures, the chemical-class probability, the ontology identifiers, the spectral identifiers, and the confidence level—these are not metadata about the annotation; they are the annotation. Reducing them to a single string (“*L-carnitine*”) and a single confidence label (“*MSI Level 2*”) is a form of information destruction that occurs at the point of laboratory output and is essentially irrecoverable downstream.

The conceptual alternative is to represent each feature as a structured evidence object. Such an object carries, at minimum, the observed m/z, the inferred neutral mass, the adduct hypothesis, the retention time (and, where possible, a retention index calibrated to a standard series), the collision cross-section where ion mobility was used, the MS/MS spectrum, the library match score, the *in silico* match score, the formula candidates with their probabilities, the structural candidates with their probabilities, the chemical-class assignment with its probability, the ontology identifiers (ChEBI, InChIKey, HMDB, PubChem), the spectral identifiers (Universal Spectrum Identifier where applicable), and the confidence level by both the MSI and Schymanski schemes (Sumner et al., 2007; Schymanski et al., 2014). The mzTab-M version 2.0 format was designed precisely to support this richer representation, with explicit slots for ambiguity, multiple identifications per feature, and evidence trails (Hoffmann et al., 2019). That format exists, but its use in published exposomics is still rare.

Why does the evidence-object view matter for meta-analysis? Because it elevates annotation confidence to a first-class statistical variable, rather than treating it as a footnote. A Level 1 standard-confirmed metabolite, a Level 2 spectral-library match, a Level 3 tentative candidate, a Level 4 unequivocal molecular formula, a Level 5 mass of interest, a CANOPUS class assignment, and a recurring unknown with a USI are all valid pieces of evidence. They are not equivalent, and meta-analysis should not assume they are. With evidence objects, a meta-analyst can stratify analyses by confidence: pool only Level 1 and Level 2 across studies; perform a separate class-level meta-analysis for CANOPUS-annotated features; conduct a third meta-analysis on recurring unknowns matched by spectral similarity rather than by name; and report each stratum separately. Without evidence objects, all annotations are reduced to a single string and a single column, forcing the meta-analyst to choose between discarding low-confidence rows (which biases the analysis toward known chemistry) or pooling them with high-confidence rows (which contaminates the analysis with false positives). With evidence objects, this choice can be made transparently, stratum by stratum, rather than at the meta-analysis stage.

Unknowns deserve special attention. The instinct of conventional meta-analysis is to discard them. The instinct of exposome science should be the opposite, as unknowns represent the discovery space. A feature that consistently appears in many samples, with a stable retention time, a stable MS/MS spectrum, and no library match, is exactly the kind of signal that a non-targeted screen is designed to reveal. If it correlates with a phenotype across multiple studies, that is a finding, even before it has a name. The Universal Spectrum Identifier (Deutsch et al., 2021) makes such cross-study citation technically straightforward: each laboratory deposits the MS/MS spectrum into a participating repository, the spectrum receives a USI, and downstream analyses can cite the unknown by its USI instead of an arbitrary local name. ReDU and the recent pan-repository reanalysis infrastructure enhance this even further, as spectral similarity searches across all deposited tandem spectra can group features from different laboratories that have different local labels (Hosseini et al., 2025; Jarmusch et al., 2020). With these tools, a “*recurring unknown*” can be treated as a stable, citable, statistically tractable entity in a meta-analysis, even before its structure is resolved.

It is worth being specific about what this implies for reporting practice. A meta-analysis-ready untargeted exposomics study should not only deposit raw data and feature tables but also, for each feature, a structured record that includes the evidence object I have just described. The mzTab-M format already provides the schema; the work for the field is to make its use routine, to write validators that flag missing evidence fields, and to require repositories to enforce a minimum subset before accepting a deposition. The choice between “*we ran a non-target study and got a list of compounds*” and “*we ran a non-target study and produced a structured catalog of features with evidence*” is the choice between an island and a building block. Exposome science requires building blocks.

There is a deeper point underlying this. The MSI and Schymanski schemes were originally proposed as confidence levels for the identification of individual compounds in individual studies, to standardize how scientists communicate the strength of their assignments. They have done that admirably. What they did not anticipate, because few standards foresee it, is that confidence levels would later need to function as statistical weights in cross-study evidence synthesis. The next decade of work on annotation should focus on this second role: confidence not as a label for a paper to display, but as a statistical input to a meta-analytic estimator that can combine high-confidence identifications, putative annotations, class-level inferences, and recurring unknowns into a single estimate of association with an exposure or phenotype, with calibrated uncertainty. That work has barely begun, and it is one of the most important methodological challenges the field faces. Tidy-data principles applied to exposomics data structures, as implemented in tidyexposomics (Laird et al., 2026), provide one concrete model for representing exposure, omics, and codebook metadata in a form that supports downstream confidence-stratified meta-analysis.

## 7 The quantification problem and a five-tier scheme

If annotation is the most important conceptual problem in untargeted exposomics, quantification is the most important numerical one. The two are related but separable. A feature may be confidently annotated and still poorly quantified; a feature may be well-quantified, in the sense of yielding a stable intensity, and still poorly identified. Meta-analysis requires that both be addressed. We have recently described some approaches for semi-quantification of untargeted metabolomics (Sillé et al., 2026a,b), which is often sufficient, especially when applying threshold of toxicological concern (TTC) thinking (Hartung, 2017).

The basic difficulty is that untargeted high-resolution mass spectrometry does not, in its native operation, return concentrations. It returns peak areas. The relationship between peak area and concentration depends on ionization efficiency, matrix effects, instrument response, integration parameters, and the calibration approach (Myint et al., 2017). For a small number of features in any given study, an authentic chemical standard has been run alongside the samples, a matrix-matched calibration curve has been constructed, and an absolute concentration in nanomolar or nanograms per milliliter has been derived. For the great majority of features, this is not the case, and the reported intensity is some form of normalized peak area whose absolute scale is not directly interpretable. NORMAN's guidance has surveyed the resulting quantitative challenges in detail (Hollender et al., 2023), and BP4NTA has developed a parallel set of recommendations for non-target analysis performance metrics, including accuracy, uncertainty, and error sources (Fisher et al., 2022; Black et al., 2023).

**Figure 5:**
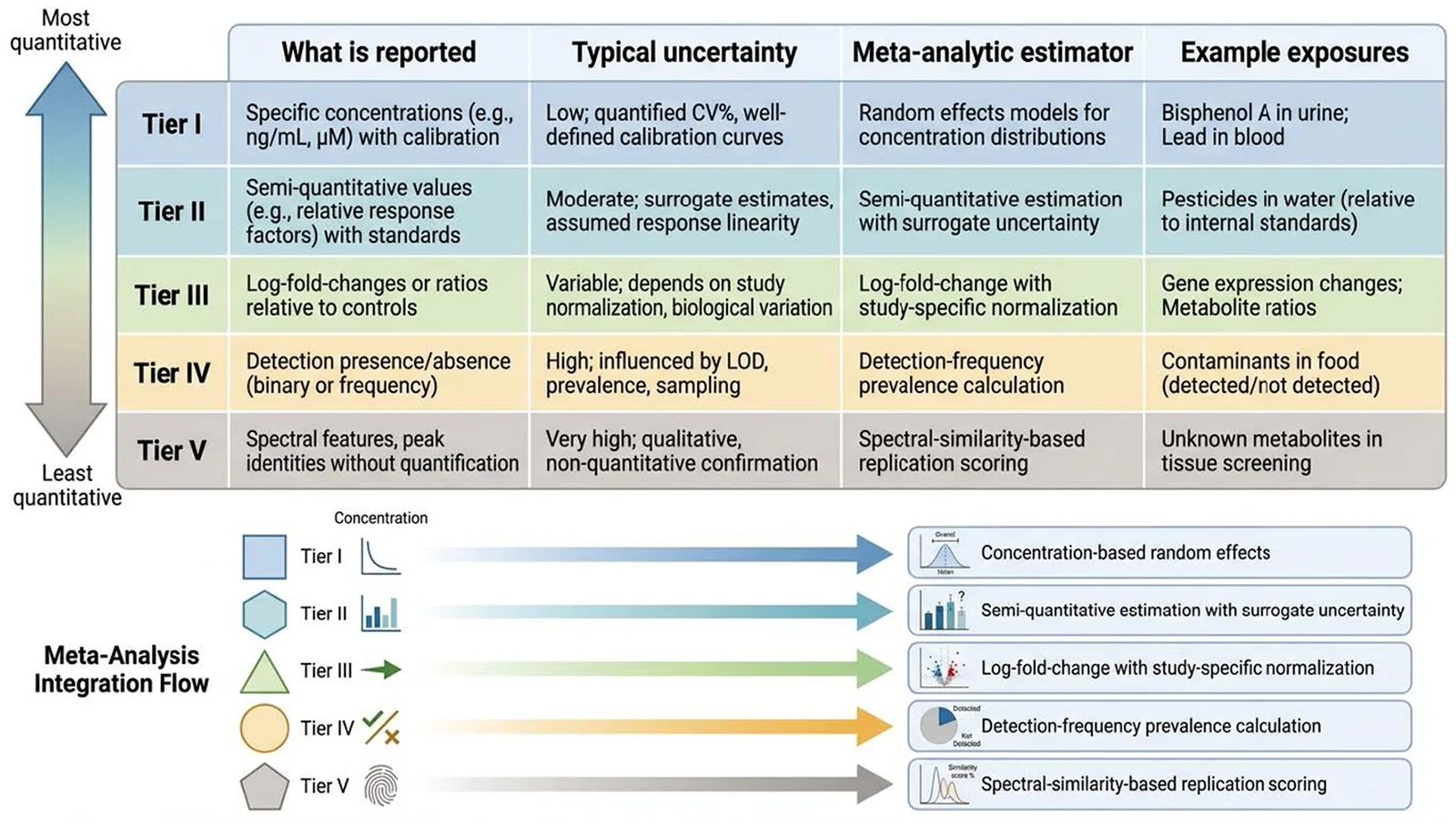
The five-tier quantification scheme. Untargeted metabolomics intensities vary in how directly they reflect concentration. The figure proposes a five-tier scheme that stratifies features by their quantitative interpretability: Tier I (absolute quantification with authentic standards and matrix-matched calibration); Tier II (semi-quantification with surrogate standards); Tier III (intensity ratio relative to internal standards or pooled QC); Tier IV (presence/absence with detection frequency reporting); Tier V (unknown feature evidence). Each tier supports a different downstream meta-analytic estimator. The figure shows, for each tier, what is reported, the typical uncertainty, the appropriate meta-analytic estimator, and example exposure categories for which the tier is typical.

What can be done about this for meta-analysis? I do not think the answer is to demand absolute quantification of every feature, because that would defeat the purpose of an untargeted approach. The answer is to be transparent about the type of quantification that was actually performed and to stratify meta-analytic estimators accordingly. This article proposes the following five-tier scheme (Figure 5), which I have used in my own thinking and which I find clarifying enough that I am willing to publish it as a starting point for community discussion:

Tier I: Absolute quantification with authentic standards and matrix-matched calibration: A concentration in mass per volume or mole per volume, with documented limits of detection and quantification, and a calibrated uncertainty. This is the gold standard, achievable for tens to a few hundred features per study with current effort, and it is the only quantification that supports dose–response inference and direct cross-study comparison.

Tier II: Semi-quantification with surrogate standards: A concentration estimated from authentic standards for chemically similar but not identical compounds, or from class-based response factors. Uncertainty is larger than Tier I but bounded, and meta-analysis can incorporate the surrogate-based uncertainty as part of the standard error.

Tier III: Intensity ratio relative to internal standards or pooled QC: A normalized peak area expressed as a ratio. This supports cross-sample comparison within a study and, with care, log-fold-change meta-analysis across studies that share an internal standard scheme. It does not support cross-study comparison of magnitude.

Tier IV: Presence/absence with detection frequency reporting: A binary call per feature per sample, with documented thresholds. This is appropriate for low-abundance environmental contaminants where intensity is too unreliable to support a continuous estimator, but detection frequency is itself biologically meaningful.

Tier V: Unknown feature evidence: A feature without a confirmed quantitative interpretation, characterized by its spectral identifier, retention behavior, and presence in samples. This tier is included to ensure that recurring unknowns are not silently dropped, allowing meta-analysis to report their cross-study replication, even if their absolute or relative magnitude is not known.

The purpose of the scheme is not to dictate which tier is correct for a given exposure. Instead, a study should report which tier applies to each feature, enabling the meta-analyst to stratify accordingly. A relative-intensity association is genuinely useful, but it is not a concentration–response relationship, and the field has been too tolerant of language that obscures this distinction. When two studies report a “*two-fold increase*” in some metabolite, they may be describing 2-fold increases in different contexts—one a true concentration and the other a normalized peak-area ratio whose underlying response factor is unknown. The five-tier scheme makes this distinction explicit. The mzTab-M format provides slots for the necessary metadata (Hoffmann et al., 2019), and an explicit “*quantification tier*” column in the feature table would be a low-cost, high-information addition to the existing schema.

For meta-analysis, the practical use of the scheme is straightforward. A Tier I meta-analysis pools studies that achieved absolute quantification for a given analyte and proceeds with classical random-effects estimation of effect sizes on the concentration scale, appropriately handling detection limits. A Tier III meta-analysis pools studies that produced relative intensities, understanding that the underlying scale is not comparable across instruments, with the appropriate estimator on the log-fold-change scale and study-specific normalizations as random effects. A Tier IV meta-analysis pools detection frequency data and produces a population-level prevalence estimate per analyte. A Tier V meta-analysis pools recurring unknowns and generates a cross-study replication score. The estimators are different; the contributing studies are different; the interpretations are different. By explicitly stratifying, the meta-analyst avoids forcing studies of one tier into the inferential frame appropriate for another, allowing the reader to quickly understand what kind of evidence is being combined.

There is one further consideration that deserves mention here. Absolute quantification, for some exposure categories, is now within reach for many more features than the field has historically treated as absolutely quantified. Calibration approaches that use a small set of internal standards together with predicted response factors derived from molecular descriptors are beginning to provide concentration estimates for hundreds of features per study, with documented uncertainties (Loos and Singer, 2017). These approaches are noisier than authentic-standard calibration, but they are far more informative than relative intensity alone and should be treated as a Tier II option in the scheme above. The field should encourage their use and report results in a comparable form.

## 8 What meta-analysis currently can and cannot do

**Figure 6:**
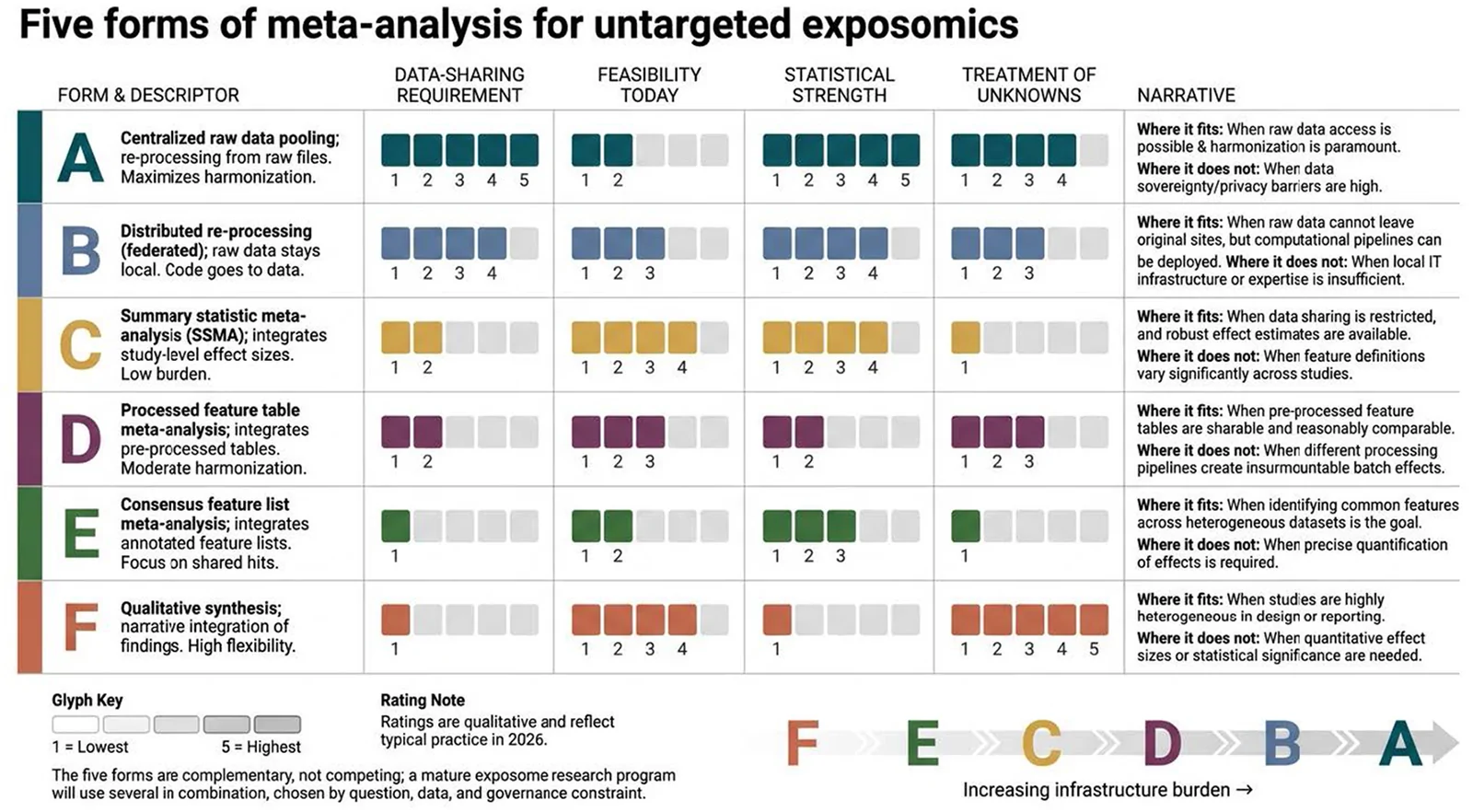
Five forms of meta-analysis for untargeted exposomics. Meta-analysis of untargeted metabolomics-based exposomics is not a single technique but a portfolio of five (or six) practical forms, each with characteristic strengths, weaknesses, and feasibility under current infrastructure—they are graded by the author's judgment. Form A: Raw data pooled reprocessing (gold-standard pooling of raw spectra under a single pipeline). Form B: Processed-feature harmonization (mapping processed features across studies via m/z, retention time, MS/MS, CCS, and USI). Form C: Metabolite-level summary-statistic meta-analysis (random-effects or Bayesian estimation across per-study effect sizes for harmonized analytes). Form D: Chemical class, pathway, or molecular family meta-analysis (aggregation at the class or network level when compound-level identifications diverge). Form E: Federated meta-analysis (per-cohort local analysis with export of only summary statistics, suitable for governance-constrained cohorts). Form F: Detection frequency meta-analysis (presence/absence aggregation across studies; included here as a primitive but informative special case). Each form is rated along four axes: data-sharing requirement (low to high), feasibility today (immediate to aspirational), statistical strength (qualitative to quantitative effect estimation), and treatment of unknowns (discards them to first-class evidence). The color-coded matrix highlights, at a glance, that no single form dominates and that a mature exposome research program will use several in combination, chosen by question, data, and governance constraint.

With a richer conception of annotation and a stratified conception of quantification, what kinds of meta-analysis are actually feasible today? Five practical forms can be identified, each with characteristic strengths, weaknesses, and feasibility today (Figure 6). The five are not mutually exclusive; a mature exposome research program will use several of them in combination.

### 8.1 Form A: raw data pooled reprocessing

This is the gold-standard form of meta-analysis. Raw data, including blanks, quality control injections, and acquisition metadata, are obtained from each participating study, ideally from a public repository under FAIR terms, and reprocessed together using a common pipeline. Peak picking, retention time alignment, blank subtraction, batch correction, annotation, and statistical modeling are all performed using the same software, parameters, and decisions. The result is a single feature-by-sample matrix with internally consistent processing, on which conventional statistical analyses can be conducted. The major recent advance making this approach more feasible is pan-repository reanalysis infrastructure, which allows queries across MetaboLights, Metabolomics Workbench, and GNPS/MassIVE (see text footnote 1) to be executed under harmonized metadata (Hosseini et al., 2025).

The strength of this approach is that it eliminates almost all sources of cross-study heterogeneity because all sources are addressed at the processing stage. The weakness lies in scalability and ecological validity. Studies that used different chromatography, different ionization modes, different instruments, or different sample preparation cannot be processed identically without producing artifacts. Even when raw data are available, the reprocessing effort is substantial, and the resulting feature-by-sample matrix may not be appropriate for studies whose populations differ in critical ways. Raw data pooling is best suited to a small number of studies with similar platforms, similar matrices, and a common scientific question, and is the approach of choice for prospective consortium analyses where data acquisition has been deliberately coordinated.

### 8.2 Form B: processed-feature harmonization

In this approach, raw data are not pooled. Instead, each study's processed feature table is mapped onto a common feature space using m/z, retention time (where indexed to a common series), collision cross-section (where ion mobility was used), neutral mass, adducts, MS/MS spectra, and feature groupings. Universal Spectrum Identifiers can be used to anchor specific spectra. The result is a meta-feature table in which each row represents a feature identified across multiple studies, along with per-study annotations, intensities, and effect estimates.

The strength of this approach is that it can scale to studies whose raw data are not available, provided their processed outputs are reported in a machine-readable form such as mzTab-M (Hoffmann et al., 2019). The weakness is that feature matching across laboratories is probabilistic. A common m/z and a similar MS/MS spectrum provide reasonable evidence that two features represent the same chemical entity, but they do not constitute proof. Retention time alignment across instruments is particularly challenging, because chromatography conditions differ between laboratories, and indexed retention measurements are uncommon in human exposomics. We recently discussed some approaches to overcome this challenge (Sillé et al., 2025). Collision cross-section, when available, helps considerably, as it is approximately instrument-independent. Spectral library matching across studies is becoming more reliable as libraries grow, but it remains imperfect, particularly for unknown features. Processed-feature harmonization is therefore an approach that works well for confidently annotated features and degrades gracefully for putative and class-level annotations.

### 8.3 Form C: metabolite-level summary-statistic meta-analysis

This is the form of meta-analysis most directly analogous to clinical-epidemiology practice. Each study reports, for a defined set of harmonized analytes or annotations, an effect estimate (e.g., a beta coefficient from a linear model) and a standard error, along with the model specification, covariate set, and transformation applied. The meta-analyst combines these per-study estimates using random-effects, fixed-effects, or Bayesian hierarchical estimators (Higgins et al., 2003), with false discovery rate control across the set of analytes considered.

The strength of this approach is that it is statistically tractable, well-understood, and compatible with restricted data sharing. It is also the most realistic option today for cohort studies governed by data protection regulations that preclude raw data sharing. The weakness is that it requires harmonization at the analyte level, which discards unknowns and lower-confidence features, and depends on each study having performed a comparable statistical analysis with a comparable covariate set. A meta-analysis-ready exposomics study should produce, as a routine output, a summary-statistic export in a structured format that can be ingested by downstream meta-analytic software. No such export schema is widely adopted today, although the components exist within mzTab-M and ISA.

### 8.4 Form D: chemical class, pathway, or molecular family meta-analysis

When compound-level identifications are uncertain, meta-analysis can be conducted at a higher level of chemical organization: chemical classes (e.g., per- and polyfluoroalkyl substances, organophosphates, polycyclic aromatic hydrocarbons), lipid classes, pathway modules, or molecular network communities. CANOPUS class assignments (Dührkop et al., 2021), ChEBI ontology lookups (Hastings et al., 2016), pathway databases such as KEGG and Reactome, and molecular network communities defined by feature-based molecular networking (Nothias et al., 2020) all support this kind of aggregation.

The strength of this approach is that it can pool studies whose feature-level annotations diverge, because the class is a coarser unit that more studies can agree on. It is also biologically informative: many exposomic associations are class effects rather than single-compound effects, particularly for mixtures. The weakness is the loss of specificity. A class-level association may be biologically meaningful but less actionable for exposure mitigation or regulation. A finding that “*the PFAS class is elevated in cases vs. controls*” is useful, but it does not inform a regulator which PFAS to act on.

### 8.5 Form E: federated meta-analysis

For cohort studies governed by privacy, consent, or biobank restrictions, raw data cannot leave their home institution. Meta-analysis in such contexts must be federated: each participating cohort performs the analysis locally, exporting only summary statistics, model outputs, or aggregated counts to a central analyst. The DataSHIELD platform and similar federated analysis frameworks have demonstrated the technical feasibility of this approach in genetic epidemiology (Gaye et al., 2014), and the GA4GH human exposome data standards effort is explicitly intended to enable federated exposome analyses by harmonizing the variables that each cohort exposes (GA4GH Human Exposome Data Standards Study Group, 2024).

The strength of this approach is that it can include cohorts that cannot otherwise share data, which applies to most large human exposome cohorts. The weakness is that federation only works if participating studies have harmonized their variables, models, and exports in advance. A federated analysis launched after the fact, involving cohorts that did not coordinate, typically fails because the variables exposed by each cohort are not actually comparable. Federated analysis therefore depends on the very interoperability profile this article advocates for, more than any other form. Federation also unlocks a class of AI workflows that would otherwise be impossible. Modern federated learning architectures train a global predictive model across multiple cohort sites by exchanging only model parameters, gradients, or aggregated summary statistics—never raw data—between local nodes and a central coordinator (Rieke et al., 2020; Kairouz et al., 2021). For exposomics, the relevant primitive is the Layer 4 schema: when every cohort exports its effect estimates, standard errors, model specifications, and covariate sets in a harmonized form, those exports provide precisely the gradients and sufficient statistics a federated learner needs. Cohorts that cannot share raw plasma metabolomics data due to GDPR, biobank governance, or consent restrictions can nevertheless contribute to a global model of exposure–disease association by exporting only Layer 4 outputs. Differential privacy noise can be added to those outputs to make individual-level reconstruction provably difficult (Dwork and Roth, 2014). The result is a single AI model of the chemical exposome that has been trained on the union of cohorts that could not have been pooled centrally, representing the operational realization of holistic data fusion in the sense of Exposome Intelligence (Hartung, 2025). This is the form of meta-analysis that scales to the human exposome.

Because federated learning (Luechtefeld and Hartung, 2025) is likely to become the dominant mode of large-scale exposome modeling, its adoption deserves more comprehensive treatment than a single paragraph. Several practical dimensions must be addressed. Model governance requires an agreed protocol for who trains, updates, and validates the global model, and for how model versions are documented and audited across participating sites. Privacy protection relies not only on keeping raw data local but also on limiting what the exchanged parameters or summary statistics can reveal; differential privacy noise (Dwork and Roth, 2014) and secure aggregation reduce, but do not eliminate, reconstruction risk, and the residual risk should be quantified rather than assumed away. Model bias is a primary concern because if the contributing cohorts are geographically or demographically skewed, the federated model inherits that skew, and harmonization at Layer 4 does not, by itself, correct it. Computational requirements are substantial since each site must run a comparable local pipeline and maintain the infrastructure to participate in training rounds. Harmonization remains the critical constraint: federated learning presupposes that each cohort exposes comparable variables, models, and covariate sets, which is precisely the interoperability the four-layer stack is designed to provide. These are not hypothetical obstacles. They have been confronted in existing federated biomedical AI initiatives, from DataSHIELD in genetic epidemiology (Gaye et al., 2014) to multi-institutional clinical models such as the EXAM study, which trained a federated predictor of COVID-19 outcomes across 20 hospitals worldwide without centralizing patient data (Dayan et al., 2021), and in the advances and open problems cataloged for the field more broadly (Rieke et al., 2020; Kairouz et al., 2021). Exposomics should learn from these precedents rather than rediscover their lessons.

### 8.6 Form F: detection frequency meta-analysis (a sixth form, in passing)

This is a special case worth naming explicitly. Across studies, the simple fact that a given chemical entity is detected at all is informative. The presence or absence of a feature across cohorts, populations, and matrices is a primitive form of evidence that does not require quantitative comparison and scales straightforwardly across thousands of studies. NORMAN's Suspect List Exchange (Taha et al., 2022) has demonstrated the value of presence/absence aggregation for environmental monitoring, and an analogous framework for human exposomics would be valuable.

The five (or six) forms are not in competition. A mature exposome research program will use them in combination, choosing the form appropriate to the question, the data, and the governance constraints. The point of enumerating them here is to clarify that meta-analysis of untargeted exposomics is not a single technique. It is a portfolio. The interoperability profile this article proposes should support all of them.

## 9 An interoperability profile: the exposome–metabolome evidence stack

**Figure 7:**
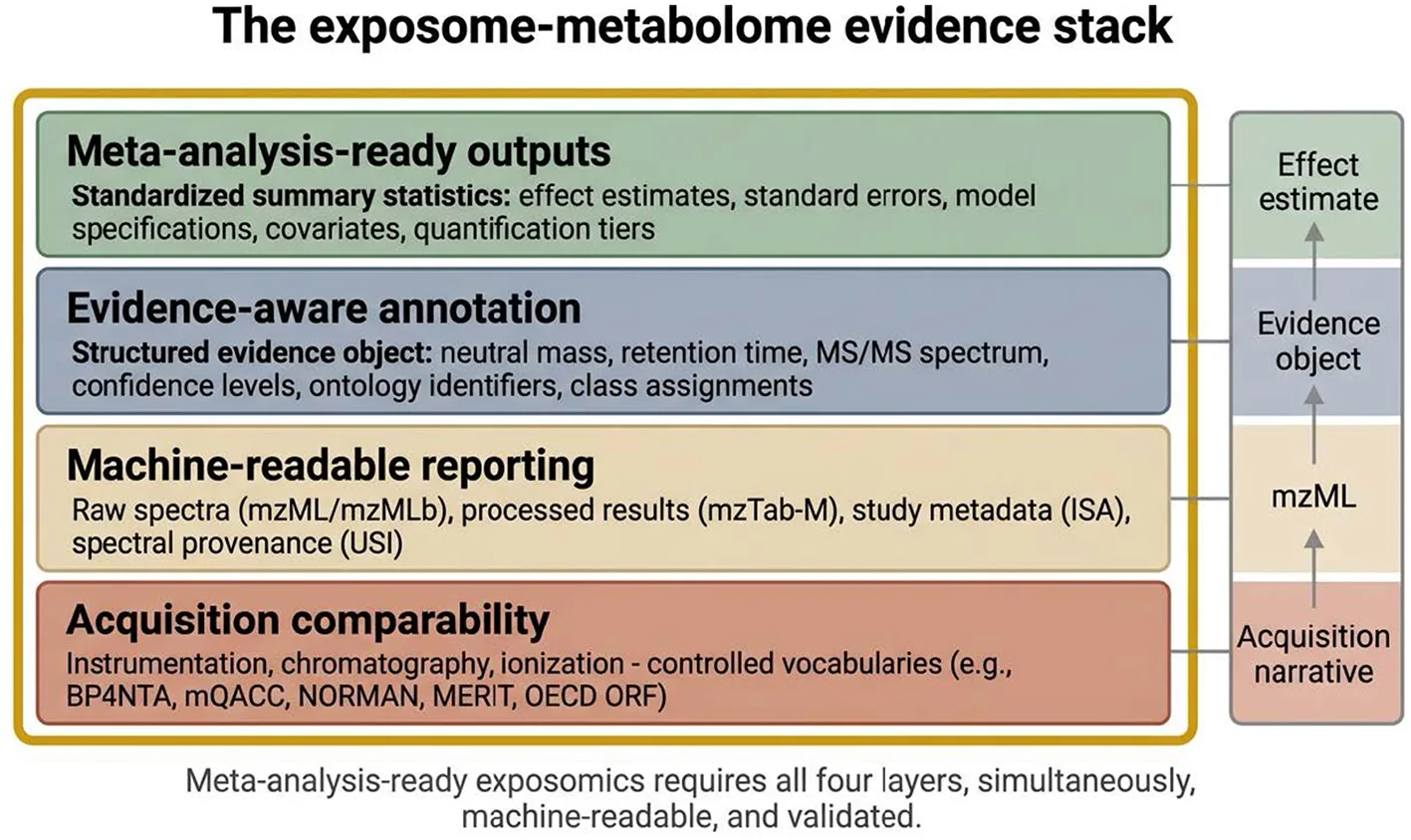
The exposome–metabolome evidence stack (central conceptual figure). The exposome–metabolome evidence stack is a four-layer interoperability profile for untargeted exposomics meta-analysis. Layer 1 (acquisition comparability) ensures that differences in instrumentation, chromatography, and ionization are explicitly reported using a controlled vocabulary. Layer 2 (machine-readable reporting) ensures that raw spectra (mzML/mzMLb), processed results (mzTab-M), study metadata (ISA), and spectral provenance (USI) are deposited in open, validatable formats. Layer 3 (evidence-aware annotation) represents each feature as a structured evidence object carrying neutral mass, retention time, MS/MS spectrum, confidence levels, ontology identifiers, and class assignments. Layer 4 (meta-analysis-ready outputs) exports standardized summary statistics (effect estimates, standard errors, model specifications, covariates, quantification tiers) for direct ingestion by downstream meta-analytic software. Each layer is necessary; none is sufficient.

This article proposes, as its central conceptual contribution, a four-layer evidence stack for untargeted exposomics, designed to be implemented by existing standards working groups rather than to compete with them. The layers correspond to four distinct kinds of interoperability, each of which is necessary, and none of which is sufficient on its own. The stack is shown schematically in Figure 7.

### 9.1 Layer 1: acquisition comparability

The first layer does not focus on standardizing acquisition. Untargeted exposomics requires chemical breadth, and forcing every study into a single platform would defeat that purpose. What the field needs is comparable reporting of acquisition: sufficient detail, in a machine-readable form, to make differences explicit and to allow downstream analyses to stratify or pool appropriately. The relevant existing standards include the t^4^ quality-assurance workshop framework that anticipated mQACC (Bouhifd et al., 2015) and mQACC's subsequent work on quality assurance, community practice, and reference materials in untargeted metabolic phenotyping (Beger et al., 2019; Evans et al., 2020; Lippa et al., 2022), BP4NTA for environmental non-target analysis (Place et al., 2021; Peter et al., 2021), NORMAN's guidance on suspect and non-target screening (Hollender et al., 2023), and MERIT (see text footnote 8) plus the OECD Omics Reporting Framework for regulatory contexts (Viant et al., 2019; OECD, 2023). These overlap substantially. The task for the field is to produce a crosswalk that identifies, for each acquisition parameter, which standard's vocabulary should be used and where the validators reside.

### 9.2 Layer 2: machine-readable reporting

The second layer involves converting the standard methods narrative into a structured, machine-readable representation. The relevant existing standards include mzML and mzMLb for raw mass spectrometry data (Martens et al., 2011; Bhamber et al., 2021), mzTab-M for processed metabolomics results (Hoffmann et al., 2019), ISA-Tab and ISA-JSON for study, sample, and assay metadata (Sansone et al., 2012), USI for spectral provenance (Deutsch et al., 2021), and repository accessions (MetaboLights, Metabolomics Workbench, GNPS/MassIVE) for the provenance of the deposited data themselves (Yurekten et al., 2024; Sud et al., 2016; Wang et al., 2016). The goal for the field is to require these formats not as optional but as the operational substrate of every untargeted exposomics study, with validators run at the repository as a precondition for acceptance.

### 9.3 Layer 3: evidence-aware annotation

The third layer is the evidence-object view of annotation discussed in Section 6. The relevant existing standards include the MSI confidence levels (Sumner et al., 2007) and the Schymanski confidence levels (Schymanski et al., 2014) for identification confidence; the GNPS molecular networking and feature-based molecular networking infrastructure for evidence aggregation across spectra (Wang et al., 2016; Nothias et al., 2020); SIRIUS, CSI:FingerID, CANOPUS, and MSNovelist for structural and class-level annotation beyond direct library matches (Dührkop et al., 2019, 2021; Hoffmann et al., 2022); RefMet for nomenclature normalization (Fahy and Subramaniam, 2020); ChEBI and InChIKey for chemical identity (Hastings et al., 2016; Heller et al., 2015); and USI for spectral identity (Deutsch et al., 2021). The goal for the field is to converge on a structured evidence-object schema that integrates these elements: a single record per feature, carrying confidence levels, identifiers, and the evidence behind them.

### 9.4 Layer 4: meta-analysis-ready outputs

The fourth and most consequential layer is the production of a standardized summary-statistics layer for every untargeted exposomics study. The schema should include, for each feature or annotation: an effect estimate, a standard error, the model specification, the covariate set, the data transformation applied, the handling of missingness, the detection frequency, the quality control flags, the annotation confidence, and the quantification tier from Section 7. With these fields harmonized, random-effects meta-analysis, class-level enrichment, federated exposome-wide association, and replication analysis become feasible without raw data pooling. The relevant existing standards include mzTab-M for the feature-level fields and ISA for the study-level fields. There is no widely adopted schema for the summary-statistic export today, but the components exist, and a community working group could produce a draft schema within a year. Early operational steps in this direction are already appearing in open-source software. The tidyexposomics R package (Laird et al., 2026), developed within our group at Johns Hopkins based on tidy-data principles, provides an end-to-end exposure-omics pipeline with built-in support for ontology mapping, sample-level quality control, exposure–outcome association, exposure-wide association studies (ExWAS), differential abundance analysis, multi-omics integration, and network analysis from a single MultiAssayExperiment-style input. Tools of this kind are precisely the operational foundation the Layer 4 schema needs, as they convert the export of meta-analysis-ready summary statistics from a per-laboratory custom script into a default behavior. Tidyexposomics is freely available^30^ with example data deposited.^31^

The novelty of the proposal is not in any individual layer (Table 1). Each layer is populated, at least partially, by existing standards. The novelty lies in the requirement that all four layers be present simultaneously, that they be machine-readable, that they reference each other through stable identifiers, and that validators be run at the point of deposition. Currently, exposomics studies typically populate one or two layers. Acquisition reporting is reasonably common; raw data deposition is increasingly common; structured annotation is rare; and structured summary statistics are essentially absent. The proposal is that all four be required together before a deposition is accepted as meta-analysis-ready.

**Table 1:** What already exists vs. what this article proposes.

Component	Existing validated standard(s)	Current community adoption	Key existing limitation	Newly proposed here	Expected future development
Acquisition reporting (Layer 1)	MSI; mQACC; BP4NTA; NORMAN; MERIT; OECD OORF	Partial; vocabularies overlap and diverge	No crosswalk; terms not machine-comparable	Controlled, machine-readable acquisition crosswalk across the existing standards	Consensus crosswalk maintained by a governing body
Raw and processed data formats (Layer 2)	mzML, mzMLb; mzTab-M v2.0 (HUPO-PSI)	Raw deposition common; mzTab-M rare in exposomics	Processed results seldom shipped in mzTab-M	mzTab-M with full evidence objects required at deposition	Repository-side validators enforce format on ingest
Study and sample metadata (Layer 2)	ISA-Tab/ISA-JSON; GA4GH HEDS; Phenopackets	Used by MetaboLights; uneven cohort metadata	Cohort covariates rarely controlled-vocabulary	Harmonized covariate reporting tied to the profile	Federated-ready, phenotype-linked metadata schemas
Annotation and confidence (Layer 3)	MSI and Schymanski levels; GNPS; SIRIUS/CANOPUS; RefMet; ChEBI; USI	Levels widely cited; evidence rarely shipped structured	Annotation flattened to a name + a level	Feature-level evidence object; confidence as a statistical weight	Confidence-stratified meta-analytic estimators
Quantification (five-tier scheme)	Peak-area/QC normalization; NORMAN quantitative guidance	Semi-quantitative practice dominates	Tier of quantification usually unstated	Explicit five-tier quantification label per feature	Tier-stratified pooling; broader Tier I/II calibration
Meta-analysis-ready outputs (Layer 4)	Components latent in mzTab-M and ISA; tidyexposomics	Essentially absent as a structured export	No adopted summary-statistics schema	Standardized Layer 4 summary-statistics export schema	Community schema enabling federated meta-analysis
Cross-study demonstration	Pan-ReDU; Human Toxome Collaboratorium; analog benchmarks (CASP, CAMI)	Pan-repository reanalysis newly feasible	No exposomics meta-analysis benchmark exists	Exposomics Meta-Analysis Living Benchmark (EMLB)	Living scoreboard with pre-defined success metrics

This table separates, for each element of the interoperability profile, the existing validated standard(s) it builds on, the current level of community adoption, the main remaining limitation, the component newly proposed in this article, and the expected development to follow. It is intended as an at-a-glance integration of the proposed framework with the existing standards landscape, complementing Figures 2, 7. Adoption levels are the author's qualitative judgment.

I want to be explicit about what implementation would look like in practice. A laboratory completing a study would deposit raw spectra in mzML to MetaboLights or Metabolomics Workbench; its processed results in mzTab-M, with each feature carrying a structured evidence record according to the Layer 3 schema; its study, sample, and assay metadata in ISA; its spectral provenance in USI references; and a structured summary-statistic export according to the Layer 4 schema. The repository would run validators against each layer at submission. The deposition would receive a single accession that resolves to all four layers. A meta-analyst would query the repository for studies whose Layer 4 outputs match the analytic question, perform the meta-analysis, and stratify by Layer 3 confidence and the quantification tiers from Section 7. The entire workflow would be reproducible, auditable, and scalable.

**Figure 8:**
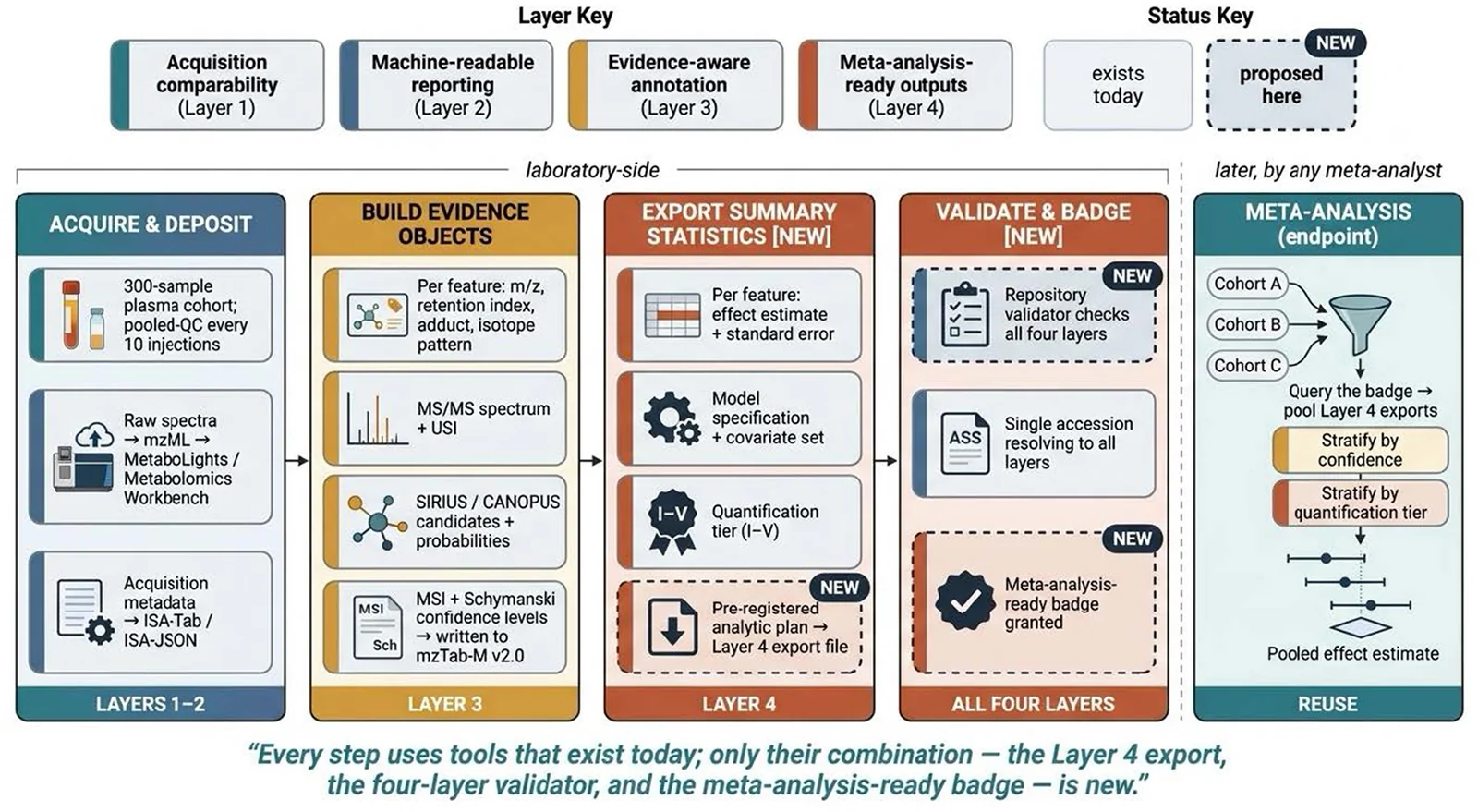
Implementing the interoperability profile: one laboratory's path from an LC-MS run to meta-analysis. A schematic of the worked example: A single untargeted metabolomics study proceeds left to right through four stages, each mapped to the evidence-stack layers by color. In Stage 1, the laboratory acquires data with pooled quality control injections and deposits raw spectra (mzML) and acquisition metadata (ISA) in a public repository (Layers 1–2). In Stage 2, it creates a structured evidence object for every feature (m/z, retention index, adduct, MS/MS spectrum with a Universal Spectrum Identifier, SIRIUS/CANOPUS candidates with probabilities, and MSI and Schymanski confidence levels) in mzTab-M (Layer 3). In Stage 3, it exports, for each feature, an effect estimate, standard error, model specification, covariate set, and quantification tier as a Layer 4 summary-statistics file. In Stage 4, a repository validator checks all four layers and issues a single accession carrying a “*meta-analysis-ready*” badge. A later meta-analyst then queries the badge across comparable cohorts, pools the Layer 4 exports, and stratifies by annotation confidence and quantification tier to obtain a pooled effect estimate. Solid outlines mark tools and standards that exist today; dashed outlines with a “*NEW*” tag mark the three components newly proposed in this article (the Layer 4 export schema, the four-layer validator, and the meta-analysis-ready badge). The figure represents a conceptual workflow, not a validated pipeline.

To make this concrete, consider a single laboratory running a 300-sample plasma cohort on reversed-phase LC-Orbitrap (Figure 8). First, it acquires data with pooled-QC injections every ten samples and deposits the raw files in mzML to MetaboLights, capturing acquisition parameters in ISA (Layers 1 and 2). Second, it processes the data, and for each feature, writes an mzTab-M record carrying m/z, retention index, adduct, the MS/MS spectrum with a USI, SIRIUS and CANOPUS candidates with probabilities, and MSI and Schymanski confidence levels (Layer 3). Third, it fits its pre-registered exposure–outcome models and exports, for each feature, an effect estimate, standard error, model specification, covariate set, and quantification tier in the Layer 4 summary-statistics file. Fourth, the repository validator checks all four layers and issues a single accession with a meta-analysis-ready badge. A later meta-analyst then queries that badge, pools the Layer 4 exports with those of comparable cohorts, and stratifies by confidence and tier, never having to contact the original laboratory or re-acquire its data. Every step in this trace uses tools that exist today; small agentic AIs might ease this, as recently shown for *in vitro* reporting standards (GIVReSt, Mohapatra and Hartung, 2026); what is new is only that the steps are performed together and validated at deposition.

This is not science fiction. Every component exists (Table 1). What does not exist is the crosswalk, the validator infrastructure, the summary-statistic schema, and the political agreement to require all four layers at deposition. None of these are technical obstacles; all are coordination challenges. The remainder of this article addresses what would be needed to overcome them.

### 9.5 The evidence stack and Trustworthy AI: a TREAT mapping

The four-layer evidence stack is not only an interoperability profile; it is, in the language of computational biomedicine, the data substrate of Trustworthy AI (Luechtefeld and Hartung, 2026). Every property a machine learning model in Exposome Intelligence (Hartung, 2025) needs to be regulator-grade (Hartung et al., 2025), Trustworthiness, Reproducibility, Explainability, Applicability, and Transparency, jointly referred to as the TREAT principles, is impossible without an upstream substrate that supplies each property in machine-readable form. The mapping is one-to-one. Layer 1 (acquisition comparability) supplies Reproducibility: a model trained on data from heterogeneous platforms cannot be reproduced unless the platforms are reported in a controlled vocabulary. Layer 2 (machine-readable reporting) supplies Transparency: open formats (mzML, mzTab-M, ISA) make the model's inputs auditable rather than opaque. Layer 3 (evidence-aware annotation) supplies Explainability: an annotation expressed as an evidence object with confidence levels, formula candidates, and ontology identifiers clarifies the model's feature semantics, which is the precondition for *post-hoc* interpretation methods such as SHAP, LIME, or counterfactual analysis. Layer 4 (meta-analysis-ready outputs) supplies Trustworthiness and Applicability: structured effect estimates and uncertainties, along with covariate sets and pre-registered model specifications, allow downstream models to inherit calibrated uncertainty rather than fabricate it and to declare their applicability domain against the same cohort and exposure variables that defined the training data.

The implication is operational: AI in exposomics is only as good as the substrate it is trained on, and the substrate is precisely what this article proposes. A predictive model of exposure–disease association built on raw deposited data, lacking Layer 3 evidence objects and Layer 4 summary statistics, will be irreproducible, opaque, and impossible to audit against regulatory criteria, regardless of its internal architecture. A predictive model built on the evidence stack inherits, by construction, the components a regulator can interrogate. This is why the proposed profile is not optional for the deployment of Exposome Intelligence (Hartung, 2025); it is a necessity.

## 10 A reporting standard for meta-analysis-ready exposomics

If the field accepts the evidence stack, the next question is what an actual reporting standard derived from it would look like. I want to outline one, not as a final specification, but as a starting point for the working groups that should produce it. The outline (Table 2) is organized into required, recommended, and optional fields, following the convention used in MSI, MERIT (see text footnote 8), and BP4NTA (see text footnote 7) reporting standards.

**Table 2:** Required, recommended, and optional reporting fields for meta-analysis-ready untargeted exposomics.

Field	Standard or vocabulary	Tier
LAYER 1—Acquisition comparability		
Sample matrix (biofluid or tissue type)	ISA-Tab, mQACC	Required
Sample collection date and time	ISA-Tab, GA4GH HEDS	Required
Fasting status at collection	ISA-Tab, mQACC	Required
Anticoagulant or stabilizer used	mQACC, MERIT	Required
Storage conditions and time to processing	mQACC	Required
Sample extraction protocol	mQACC, BP4NTA, MERIT	Required
Internal standards used	mQACC, NORMAN	Required
Derivatization protocol (GC-MS only)	mQACC, MERIT	Required
Chromatographic column chemistry and dimensions	mQACC, NORMAN	Required
Mobile-phase composition, gradient, flow rate, temperature	mQACC, BP4NTA	Required
Injection volume and acquisition order	mQACC	Required
Ionization mode and polarity-switching strategy	mQACC, NORMAN	Required
Ion-source parameters and lock-mass strategy	mQACC	Required
Mass analyzer vendor, model, and stated resolution	mQACC, HUPO-PSI mzML	Required
Acquisition mode (DDA/DIA, with explicit windowing)	mQACC, BP4NTA	Required
Blank scheme and frequency	mQACC, BP4NTA, NORMAN	Required
Pooled-QC injection scheme and frequency	mQACC	Required
Batch structure and randomization plan	mQACC	Required
Reference materials (e.g., NIST SRM 1950) used	mQACC (Lippa et al., 2022)	Recommended
Retention time indexing (Kovats or alkylphenone series)	mQACC, NORMAN	Recommended
Collision cross-section (CCS) measurements	NORMAN, BP4NTA	Recommended
LAYER 2—Machine-readable reporting		
Raw spectra deposited in mzML or mzMLb	HUPO-PSI mzML	Required
Processed feature table in mzTab-M v2.0	HUPO-PSI mzTab-M	Required
Study, sample, and assay metadata in ISA-Tab or ISA-JSON	ISA-tools (ISA-Tab/ISA-JSON)	Required
Universal Spectrum Identifier per annotated feature	HUPO-PSI USI	Required
Universal Spectrum Identifier per recurring unknown	HUPO-PSI USI	Recommended
Repository accession (MetaboLights/Metabolomics Workbench/GNPS-MassIVE)	MetaboLights, Metabolomics Workbench, MassIVE	Required
Validator-passing status per layer at deposition	HUPO-PSI/mQACC validators	Recommended
Cross-repository linkage (Pan-ReDU MS Run Identifier)	Pan-ReDU	Optional
LAYER 3—Evidence-aware annotation		
Observed m/z and inferred neutral mass per feature	mzTab-M	Required
Adduct hypothesis per feature	mzTab-M	Required
Retention time (and retention index where available)	mzTab-M, mQACC	Required
MS/MS spectrum and USI per annotated feature	mzTab-M, HUPO-PSI USI	Required
Library match score and library identifier	mzTab-M, GNPS	Required
*In silico* annotation score (CSI:FingerID, COSMIC)	SIRIUS/CSI:FingerID	Recommended
Candidate molecular formulas with probabilities	SIRIUS	Recommended
Candidate structures with probabilities	SIRIUS, CSI:FingerID	Recommended
CANOPUS chemical-class assignment with probability	CANOPUS	Recommended
Confidence level (MSI scheme, Sumner et al., 2007)	MSI	Required
Confidence level (Schymanski et al., 2014 scheme)	NORMAN/Schymanski	Required
Chemical identifiers (ChEBI, InChIKey, HMDB, PubChem)	ChEBI, InChI, HMDB, PubChem	Required
RefMet-standardized metabolite name	RefMet	Required
Detection frequency per matrix and per cohort subgroup	BP4NTA	Recommended
LAYER 4—Meta-analysis-ready outputs		
Quantification tier (I absolute/II surrogate/III ratio/IV presence/V unknown)	This article (proposed scheme)	Required
Effect estimate per feature per pre-specified phenotype	Meta-analysis schema (proposed)	Required
Standard error per effect estimate	Meta-analysis schema (proposed)	Required
Model specification (predictors, link function)	Meta-analysis schema (proposed)	Required
Covariate set used in the model	Meta-analysis schema (proposed)	Required
Data transformation applied (log, scaling, batch correction)	Meta-analysis schema (proposed)	Required
Missingness handling rule and imputation method	Meta-analysis schema (proposed)	Required
Multiple-testing correction strategy and FDR threshold	Meta-analysis schema (proposed)	Required
Cohort-level metadata (age, sex, BMI, smoking, medication)	GA4GH HEDS, ISA	Required
Pre-registered analytic plan (OSF or Zenodo)	OSF/Zenodo	Recommended
Pathway-level summary statistics	KEGG, Reactome	Optional
Network-community summary statistics (feature-based molecular networking)	GNPS FBMN	Optional
Linkage to external exposure data (residential, occupational)	GA4GH HEDS	Optional
Cross-omics linkage to transcriptomics/proteomics/genomics	Extension to ISA framework	Optional

The fields below describe the minimum, recommended, and optional reporting items that a meta-analysis-ready untargeted exposomics study should produce. Fields are grouped by the four layers of the exposome–metabolome evidence stack (acquisition comparability, machine-readable reporting, evidence-aware annotation, meta-analysis-ready outputs) and mapped to the existing standard, format, or vocabulary that defines them. The tier (Required/Recommended/Optional) is a proposal for community discussion, not a final specification. Required = should be present in every meta-analysis-ready deposition; Recommended = present where feasible, enables higher-confidence cross-study analysis; Optional = supports advanced or specialized re-analyses. “Meta-analysis schema (proposed)” refers to the Layer 4 summary-statistics export schema proposed in Section 10 of this article, which is not yet codified by an existing standards working group. The mapping of fields to existing standards is illustrative: many fields are addressed in more than one standard, and the final specification should harmonize the controlled vocabularies through an explicit crosswalk.

### 10.1 Required fields

At a minimum, every untargeted exposomics study deposited as meta-analysis-ready should report the following fields, in machine-readable form, with controlled-vocabulary entries where applicable. Sample provenance includes biofluid or tissue type, collection time, fasting status, storage conditions, time to processing, and any anticoagulant or stabilizer used. Sample preparation includes extraction protocol, internal standards used, derivatization (for GC-MS), and any enrichment or depletion steps. Chromatography includes column chemistry, dimensions, mobile-phase composition, gradient, flow rate, column temperature, and injection volume. Ionization includes ionization mode (positive, negative, or both, with a switching strategy), source parameters, and lock-mass strategy. The mass analyzer includes instrument vendor and model, resolution at a stated mass, scan range, scan rate, and acquisition mode (DDA, DIA with explicit windowing, or other). Quality control includes the blank scheme, the pooled-QC injection scheme with frequency, the internal standard scheme, and any reference materials used (e.g., NIST SRMs and the matrix-based and synthetic reference materials cataloged by mQACC; Lippa et al., 2022). Batch structure includes the number of batches, the randomization scheme, and the within-batch QC plan. The QA/QC questionnaire developed by mQACC provides the most comprehensive enumeration of the relevant practices today and is the natural starting point for the required-fields specification (Beger et al., 2019; Evans et al., 2020). Cohort metadata include age, sex, BMI, smoking status, medication, and other covariates harmonized to a controlled vocabulary appropriate to the cohort.

Beyond these, the required output fields are the raw spectral data in mzML (or equivalent), the processed feature table in mzTab-M with full evidence objects per feature, the study metadata in ISA-Tab or ISA-JSON, the spectral identifiers (USI) for at least the annotated features and recurring unknowns, and the summary-statistic export per the Layer 4 schema, with effect estimates and standard errors for each feature against each pre-specified phenotype or exposure, model specifications, covariate sets, and quality control flags.

### 10.2 Recommended fields

A meta-analysis-ready deposition would be more useful with the following additional fields: retention time indexing using a calibrated standard series (e.g., Kovats or alkylphenones for reversed-phase), collision cross-section measurements where ion mobility is available, authentic-standard concentrations for as many features as feasible (Tier I quantification), surrogate-standard concentrations for additional features (Tier II), library match scores and *in silico* annotation scores for each feature, CANOPUS class probabilities for unannotated features, detection frequencies per matrix and per cohort subgroup, and a pre-registered analytic plan with covariate selection, transformations, missingness handling, and multiple-testing correction strategy.

### 10.3 Optional fields

The following are valuable for advanced re-analyses but should not be required at deposition: pathway-level summary statistics, network-community summary statistics from feature-based molecular networking, linkage to external exposure data (e.g., satellite-derived air pollution, residential proximity to point sources, occupational exposure indices), microbiome-derived chemistry annotations, drug-metabolism transformation annotations, and cross-omics linkage to transcriptomics, proteomics, or genomics from the same cohort.

### 10.4 Validators

A reporting standard is only as useful as its enforcement. The most common failure mode of past standards has been the production of voluntary checklists that authors satisfy on the title page and ignore in the methods. A meta-analysis-ready standard needs validators: software that, given a deposition, returns a structured report on which required fields are present, which are missing, and which contain values outside acceptable ranges. We have recently provided an example of how small agentic AIs can check compliance with a reporting standard (Mohapatra and Hartung, 2026); similarly, agents to check compliance for this standard could be developed, possibly even importing such information from electronic laboratory notebooks. The mzTab-M format already has a reference validator (Hoffmann et al., 2019). MetaboLights and Metabolomics Workbench already run validation logs (Powell and Moseley, 2023). What is missing is a single end-to-end validator that runs against all four layers of the evidence stack, returns a single pass/fail result with field-level diagnostics, and is required at the repository before a deposition is accepted as meta-analysis-ready. Producing this validator is a 1-year project for a coordinated effort across the existing repositories and standards groups.

### 10.5 Repository enforcement

The choice between optional and required at the repository level is the single biggest determinant of adoption. If meta-analysis-ready submission is optional, most studies will not opt in because the marginal effort outweighs the marginal benefit to the depositing laboratory. If it is required for at least a subset of submissions (e.g., for studies funded by major exposome programs or for studies whose authors wish to receive a particular “*meta-analysis-ready*” badge), adoption will be much higher. The pragmatic path is a tiered submission scheme, in which a baseline deposition continues to accept current practice, but a meta-analysis-ready badge is granted only when all four layers pass validation. Funders can require the badge for studies they support, and journals can require the badge for studies they accept. Over time, the badge becomes the default.

### 10.6 The modular ecosystem: why Layer 4 compliance pays

The argument so far has emphasized enforcement: validators, repository requirements, funder mandates, and a meta-analysis-ready badge. These are the sticks. The carrots deserve equal emphasis because they will drive adoption among bench scientists who have no incentive to add work for its own sake. The right mental model is the modular data ecosystem, the BioBricks analogy from synthetic biology, in which standardizing a part makes it composable with every other part in the ecosystem and therefore reusable, citable, and consequential beyond its origin study. A Layer 4 export is, in this sense, a BioBrick for exposomics (Gao et al., 2025). The moment a study produces one, it stops being an island and becomes a node in a global, executable network. Its features can be queried in pan-repository searches such as Pan-ReDU (Hosseini et al., 2025). Its summary statistics can be ingested by any subsequent meta-analytic pipeline without manual re-extraction. Its annotations can be cited by Universal Spectrum Identifier across studies it had no contact with, including studies that did not yet exist when it was published. The carrot is not virtue; it is amplification. A meta-analysis-ready deposition multiplies the citation half-life and the public health reach of a single laboratory's work. Over time, adoption follows that math, not the mandate.

### 10.7 Adoption challenges and practical constraints

Enforcement and incentives will not, on their own, dissolve the practical constraints that determine whether a meta-analysis-ready profile is actually adopted, and a balanced proposal should outline them clearly. Laboratory resource limitations are the most immediate: producing Layer 3 evidence objects and Layer 4 summary statistics adds analyst time and computational costs that fall on the depositing laboratory, and many groups, particularly those outside well-funded consortia, lack the personnel to manage it. Differences among instrument vendors compound the problem because raw data export, controlled-vocabulary terms, and processing software vary across the major platforms, and full interoperability at the mzML and mzTab-M levels still depends on vendor cooperation, which is inconsistent. Computational infrastructure for validators, repository ingestion, and pan-repository reanalysis must be maintained by someone, and the question of who pays for that maintenance over a decade remains unresolved. Repository compatibility is likewise incomplete: MetaboLights, Metabolomics Workbench, and GNPS/MassIVE use different metadata models, and harmonizing them is an ongoing effort rather than a completed one. Long-term maintenance and community governance, finally, are the constraints most often underestimated because standards decay without stewardship, and a profile with no governing body and no sustained funding will quickly become outdated. Acknowledging these constraints is not an argument against the profile; it is a prerequisite for designing an adoption pathway that realistically addresses who bears the cost and how it is offset by the amplification benefits described above.

## 11 The Exposomics Meta-Analysis Living Benchmark

A reporting standard, however well-designed, will not be adopted unless it can be shown to work. The field's experience with previous reporting standards is instructive. MSI was published with admirable specificity in 2007 (MSI Board Members et al., 2007); two decades later, its uptake is partial and uneven. The lesson is that a checklist published in a journal is not, by itself, an intervention. What does work is a demonstration: a worked example of cumulative meta-analysis performed under the proposed standard, with the resulting evidence and the underlying tools made publicly available. I propose such a demonstration here.

**Figure 9:**
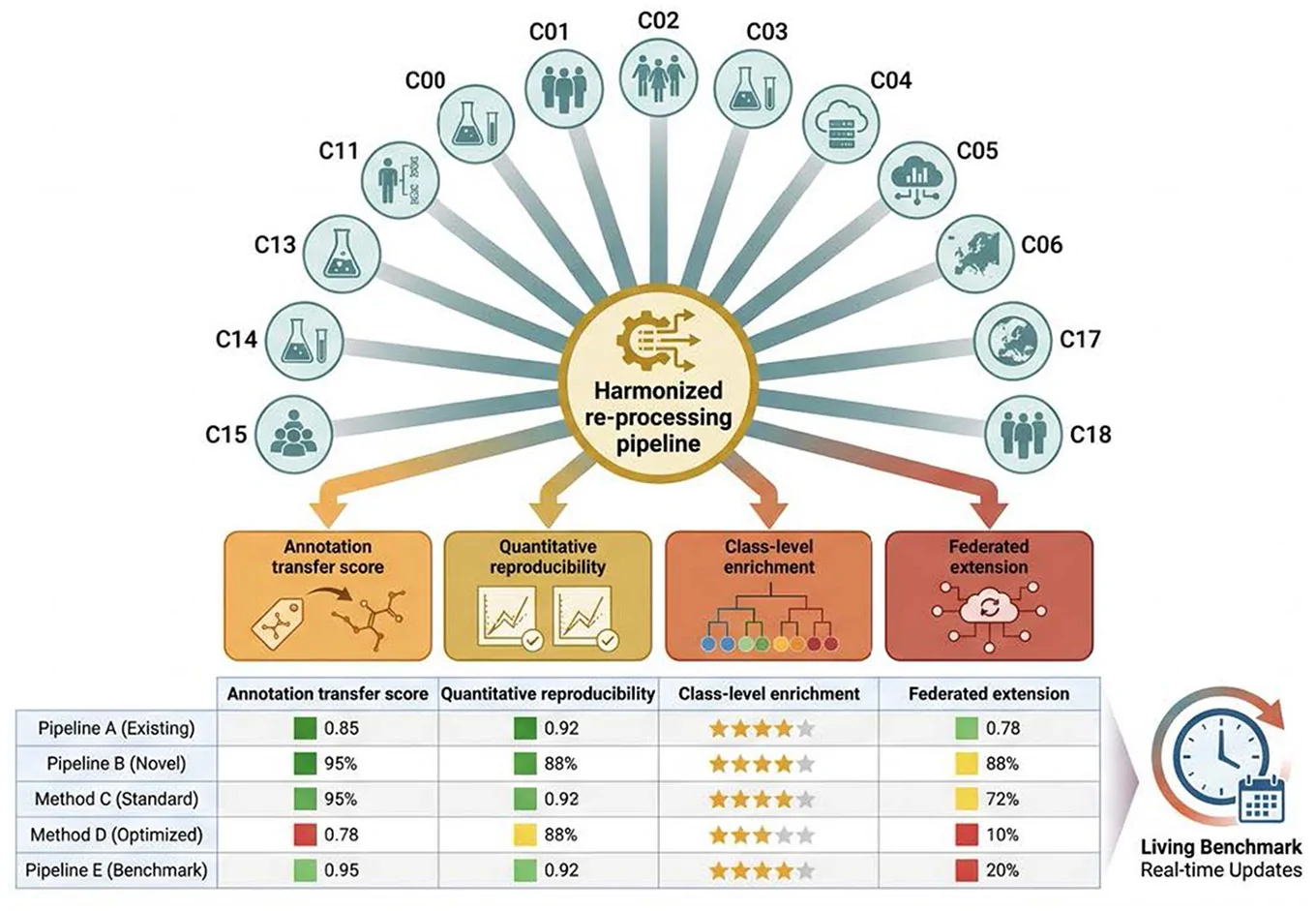
The Exposomics Meta-Analysis Living Benchmark. The Exposomics Meta-Analysis Living Benchmark (EMLB) is proposed as a community demonstration that meta-analysis-ready exposomics is feasible. A consortium would select 10–20 publicly available untargeted metabolomics studies with overlapping biospecimen types and phenotypic scopes, reprocess them under a common pipeline, and report annotation-transfer reliability, quantitative reproducibility, class-level enrichment, and federated analysis performance against a shared reference set. A public scoreboard would track performance over time as new studies, new pipelines, and new annotation engines are evaluated against the same baseline.

The proposal is for an Exposomics Meta-Analysis Living Benchmark (EMLB). The benchmark would have the following structure (Figure 9).

### 11.1 Cohort selection

A small consortium would select 10–20 publicly available untargeted metabolomics studies, drawn from MetaboLights, Metabolomics Workbench, and GNPS/MassIVE, featuring similar biospecimens (e.g., human plasma in a fasting state), broadly comparable acquisition platforms (e.g., reversed-phase LC-Orbitrap with electrospray ionization), and overlapping phenotypic scopes (e.g., obesity, type 2 diabetes, cardiovascular disease, or environmental exposures with measured external indicators). Studies would be selected explicitly to encompass a range of cohort sizes, geographic settings, and analytical platforms, ensuring that the benchmark exercises reflect rather than minimize the heterogeneity the field will encounter in practice.

### 11.2 Harmonization step

Each selected study would be reprocessed under a common pipeline. Raw data would be realigned, reannotated, and requantified using the same software, parameters, and reference libraries. Per-study summary statistics for each feature against each phenotype would be derived anew using a pre-registered analytic plan. The harmonized outputs would be deposited in a single benchmark repository, with the full provenance from raw spectra to summary statistics being machine-readable and traceable.

### 11.3 Annotation transfer assessment

For each annotated feature, the benchmark would assess agreement across studies. A confident MSI Level 1 annotation in one study should be confirmed, contradicted, or unobserved in others. Class-level annotations from CANOPUS should be replicated across studies for features whose structural identity is uncertain. Recurring unknowns identified by spectral similarity should be reported with stable USIs. The benchmark would produce, as one of its outputs, an annotation-transfer reliability score: the fraction of confidently annotated features that retain the same annotation under independent reprocessing across the benchmark cohorts.

### 11.4 Quantitative reproducibility assessment

For features with absolute quantification in multiple studies, the benchmark would assess agreement at the concentration level. For features with only relative quantification, the benchmark would evaluate log-fold-change agreement against phenotypes, using the five-tier scheme for stratification. The output would be a reproducibility surface: for each feature, the fraction of studies in which its association with phenotype replicates, stratified by quantification tier and annotation confidence.

### 11.5 Federated extension

For cohorts whose data cannot be deposited, the benchmark would offer a federated module: a packaged analytic pipeline that participating cohorts could run locally, from which they would export only the summary-statistics layer to the central analyst. This would test the federated form of meta-analysis (form E above) under realistic governance constraints.

### 11.6 Shared computational environment

Lessons can also be drawn from earlier consortium-scale efforts to provide geographically distributed teams with a common analytical workbench. The Human Toxome Collaboratorium (Fasani et al., 2016), established within the Human Toxome Project (Hartung and McBride, 2011; Bouhifd et al., 2014) and operated across six academic, commercial, and government institutions, demonstrated that a cloud-hosted virtual desktop offering a common mix of commercial, open-source, and custom-built applications can keep a geographically distributed consortium analytically synchronized, with traceable provenance, reproducible computing, and adaptability to evolving workflows. The Collaboratorium also highlighted the unique constraints that arise from running scientific computing on third-party cloud infrastructure, from data egress costs to licensing of commercial analytical software. The EMLB would inherit this framework and extend it from a single consortium to an open community resource, with the benchmark scoreboard itself hosted in such an environment so that any participating laboratory can rerun any submitted pipeline against the reference cohorts.

### 11.7 Living scoreboard

The benchmark would maintain a public, continuously updated scoreboard. New studies could be added; new processing pipelines could be evaluated against existing ones; new annotation engines could be benchmarked against the same reference cohorts. The scoreboard would not declare a single winner; rather, it would document, for each subtask (annotation transfer, quantitative reproducibility, class-level enrichment, federated estimation), the performance of competing methods on a shared, reproducible test bed. Reference pipelines such as tidyexposomics (Laird et al., under review) would be natural first entries in the scoreboard, as they already implement, end to end, several of the harmonization steps the benchmark is designed to evaluate.

### 11.8 Validation criteria and success metrics

A benchmark is only meaningful if success is defined in advance, and a central question for a Hypothesis and Theory proposal of this nature is how one would determine whether the profile is effective. The EMLB should therefore be evaluated against explicit, measurable criteria rather than general aspirations. Five criteria are proposed. First, cross-study reproducibility: the fraction of feature-phenotype associations that replicate, in direction and approximate magnitude, across independent cohorts re-analyzed under the common pipeline, stratified by quantification tier and annotation confidence. Second, feature-harmonization rate: the proportion of features in each study that can be confidently matched to a common meta-feature across cohorts by m/z, retention index, collision cross-section, and MS/MS similarity. Third, federated analysis success: whether a federated analysis executed across governance-constrained cohorts returns effect estimates statistically concordant with those from a pooled analysis of the same data where pooling is permitted. Fourth, repository-compliance metrics: the fraction of depositions passing the four-layer validator on first submission, and the median number of validator-flagged fields per deposition over time. Fifth, benchmark performance indicators: the annotation-transfer reliability score and the quantitative-reproducibility surface defined above, tracked longitudinally on the public scoreboard so that improvement can be demonstrated rather than merely asserted. Defining these targets in advance transforms the profile from an appealing idea into a falsifiable claim: if meta-analysis-ready reporting does not measurably improve these indicators relative to current practice, the proposal will have failed on its own terms.

The living benchmark would do for exposomics meta-analysis what CASP^32^ did for protein-structure prediction, what CAMI^33^ did for metagenomics, what CASF^34^ did for protein-ligand docking, and what various MS proteomics benchmarks have done for that field. It would convert a hortatory reporting standard into a substantive demonstration of cumulative science. The resources required are modest: a small consortium of contributing laboratories, a shared computing infrastructure, and an open governance model. The Pan-ReDU^35^ work has already demonstrated that the underlying repositories can support pan-repository reanalysis (Hosseini et al., 2025). The EMLB would build on that infrastructure and transform it from a technical achievement into a community institution.

I deliberately propose the EMLB as a “*living*” benchmark rather than as a one-time exercise. The exposome will not stop changing. New analytical platforms will emerge; new annotation engines will be developed; new cohorts will be added; and new phenotypes will be measured. A static benchmark would freeze the field at a particular moment in time. A living benchmark allows the field to incorporate new tools and cohorts as they arise, maintaining the same baseline reference set for continuity. This is the model of CASP and CAMI, and I see no reason exposomics should adopt anything weaker.

## 12 Regulatory and public health implications, and what to do this year

The argument so far has been methodological. This closing section connects the argument to the broader purpose of exposome science: changing how exposure-related disease is prevented, regulated, and managed in public health. The methodological infrastructure proposed in Sections 6 through 11 is not an end in itself; it is the precondition for using exposome science in regulatory toxicology, cumulative risk assessment, environmental justice, and routine public health practice.

### 12.1 Regulatory toxicology

Regulatory toxicology has been moving, slowly but visibly, toward the use of omics data in chemical safety assessment, which we refer to as toxicometabolomics (Bouhifd et al., 2013; Ramirez et al., 2013). The OECD has produced a Metabolomics Reporting Framework (OECD, 2023) within its Omics Reporting Framework^36^ intended to standardize the reporting of metabolomics and transcriptomics data for regulatory submissions (OECD, 2023). MERIT (see text footnote 8) has identified four regulatory use cases for metabolomics: derivation of points of departure via benchmark dosing, discovery of chemical mode of action, chemical grouping and read-across, and cross-species extrapolation (Viant et al., 2019). All four use cases require not just well-performed metabolomics within a single study but also the ability to combine evidence across studies, because regulatory inference relies on the consistency of effects across replicates, doses, species, and laboratories. The four-layer evidence stack and the five-tier quantification scheme are not luxuries for the regulatory pathway; they are necessities. Without them, regulatory acceptance of metabolomics data will remain limited to a handful of well-controlled industry studies, and the broader exposomics literature will not enter regulatory practice at all. With them, the literature becomes machine-readable, machine-poolable, and machine-auditable, which is the condition under which regulators can rely on it.

### 12.2 Cumulative and mixture risk assessment

The single greatest scientific deficit of current chemical risk assessment is its inability to address mixtures. A regulator who wishes to assess the cumulative risk of a class of chemicals or the combined risk of multiple co-occurring exposures has very few formal tools and little data to support those tools. Exposomics is a natural source of such data because untargeted analysis inherently captures mixtures. However, cumulative risk assessment requires class-level evidence aggregated across studies, with quantitative weights for each member of the class, mechanistic priors from *in vitro* and *in vivo* systems, and epidemiologic effect estimates from human cohorts. Each of these components exists; combining them depends on the interoperability profile this article advocates for. A class-level meta-analysis under form D, performed on the EMLB, would constitute a substantive contribution to cumulative risk assessment, particularly if linked to mechanistic priors from adverse outcome pathway frameworks (Ankley et al., 2010) and to *in vitro* NAM evidence from the Tox21 program and its successors (Krewski et al., 2020).

### 12.3 Environmental justice and disparities

A second area where meta-analyzable exposomics is important is environmental justice (Sillé et al., 2026a,b). Population-level disparities in exposure have been documented for decades for criteria air pollutants, lead, and a small number of specific contaminants. They are largely undocumented for the broader chemical exposome, simply because the data needed to document them do not exist at the relevant scale. Meta-analyses across cohorts with diverse populations, harmonized for residential exposure indices and socioeconomic variables, would produce, for the first time, exposure–disparity estimates across the actual chemical complexity of human exposure. The GA4GH (see text footnote 10) human exposome standards explicitly address external exposure variables alongside internal exposures (GA4GH Human Exposome Data Standards Study Group, 2024), and the federated form of meta-analysis is particularly suited to this question because the participating cohorts often have legal or ethical constraints on data sharing that preclude raw data pooling.

### 12.4 Global participation and the democratizing logic of the tier system

Untargeted metabolomics at the analytical state of the art described in Section 3 requires high-resolution mass spectrometry, dedicated chromatography, and trained personnel that are currently concentrated in a small number of laboratories in high-income countries. If meta-analysis-ready exposomics is defined as participation only by Tier I and Tier II laboratories, the field will replicate the same geographic concentration that has shaped most of biomedicine. This would be a failure of the Human Exposome Project (Hartung, 2023; Sillé et al., 2026a,b) before it began, because the populations most at risk from environmental exposure live disproportionately in the Global South and in low-resource settings within high-income countries. The five-tier quantification scheme proposed in Section 7 is, viewed in this light, an explicit democratization mechanism. Laboratories without authentic standards or matrix-matched calibration can still contribute Tier III intensity-ratio data, Tier IV presence/absence detection frequency data, or Tier V recurring-unknown spectral evidence, and those contributions enter the meta-analytic estimator at their appropriate weight rather than being excluded entirely. The Banbury Exposomics Consortium (Miller and Banbury Exposomics Consortium, 2025) and the Global Exposome Forum (Hartung, 2025) have stressed that the public health value of exposomic data is highest where exposure burdens are greatest, which is precisely the population for which Tier IV and Tier V data are most likely to be the only feasible measurements. A meta-analysis-ready profile that treats lower-tier data as legitimate evidence, rather than as data of inferior quality, is the technical infrastructure of an equitable global exposome science. The political infrastructure must follow.

### 12.5 Evidence-based toxicology

I have argued for two decades that toxicology should adopt the methods of evidence-based medicine, and I have helped build the Evidence-based Toxicology Collaboration to that end (Hoffmann and Hartung, 2006; Hartung, 2009a; Hartung et al., 2013b; EFSA and EBTC, 2018; Hartung and Tsaioun, 2024). Evidence-based medicine works because clinical trials produce harmonized summary statistics that can be combined in systematic reviews. Toxicology does not yet have an equivalent infrastructure, and the absence of one is a primary reason that systematic reviews in toxicology are slow, contentious, and uneven in quality. Exposomics has an opportunity to do better because it is younger and less encumbered by legacy practices. If the field adopts a meta-analysis-ready reporting standard from the start, systematic reviews of exposomic evidence will become routine. If it does not, exposome science risks reproducing the synthesis problems of conventional toxicology at a higher chemical resolution.

### 12.6 The Human Exposome Project

I argued earlier for a Human Exposome Project (Hartung, 2023) as the natural successor to the Human Genome Project. The methodological agenda of that project rests on the kind of interoperability infrastructure described here. A Human Exposome Project that funds a thousand additional untargeted metabolomics studies without coordinating their reporting will produce a thousand additional islands. A Human Exposome Project that funds the same studies under the four-layer evidence stack will produce a cumulative resource. The political and scientific case for a Human Exposome Project is, in my view, settled; the methodological case is now the limiting factor.

### 12.7 What to do this year

Big infrastructure proposals tend to drift because no individual laboratory feels the need to act. I want to close by listing concrete actions that any individual exposomics laboratory can take in 2026, without waiting for a coordinated working group to release a final specification. Each of these actions moves the field measurably closer to meta-analyzable exposomics and is feasible within current practice.

First, deposit raw data in mzML format to MetaboLights, Metabolomics Workbench, or GNPS/MassIVE as a standard default, regardless of whether the journal requires it. Second, produce processed results in mzTab-M version 2.0 rather than in laboratory-specific tab-separated files. Third, assign Universal Spectrum Identifiers to at least the annotated features and any recurring unknowns. Fourth, report confidence levels for every annotation under both the MSI and Schymanski schemes, and make the underlying evidence (m/z, retention time, MS/MS spectrum, library match score, *in silico* score, formula candidate) available for each feature. Fifth, report the quantification tier per feature according to the five-tier scheme. Sixth, export, alongside the feature table, a structured summary-statistics file following the Layer 4 schema, with effect estimates and standard errors for each feature against each pre-specified phenotype. Seventh, register the analytic plan in advance in a public repository before unblinding feature-by-phenotype associations.

None of these seven actions is technically demanding. None requires new infrastructure. Each is at the discretion of the depositing laboratory. The cumulative effect of widespread adoption, even before a finalized profile, would be substantial. If the next thousand untargeted exposomics studies all take these seven steps, the field will have transitioned from an archipelago to a continent by 2030.

## 13 Limitations and what remains to be demonstrated

A proposal of this kind should be explicit about its own limits and which of its claims are evidence-based and which remain hypotheses awaiting demonstration. The central diagnosis—that interoperability rather than analytical capability now limits cumulative exposome science—is well-supported by the existing literature and by the documented fragmentation of standards reviewed in Sections 3 through 5. The individual components of the profile, including mzML, mzTab-M, ISA, the USI, the MSI and Schymanski confidence schemes, and the public repositories, are established and validated in their own right. What remains hypothetical is the profile as an integrated, enforced whole: neither the four-layer evidence stack, the evidence-object representation, the five-tier quantification scheme, nor the Exposomics Meta-Analysis Living Benchmark has yet been implemented, benchmarked, or shown at scale to deliver the cumulative gains claimed for it, so their expected benefits are prospective rather than demonstrated.

Several concrete weaknesses deserve explicit acknowledgment. Metabolite annotation remains incomplete, so a substantial fraction of features will continue to enter meta-analysis only as class-level or recurring-unknown evidence. Laboratory and cohort heterogeneity, along with frequently missing or non-standardized metadata, will limit how many studies can be combined in practice regardless of the profile. Repositories face genuine questions of long-term sustainability and funding. Interoperability between vendor software is still imperfect. Annotation standards and spectral libraries are themselves evolving, so any fixed schema risks obsolescence and must be versioned. Additionally, uncertainty propagation across the four evidence layers—from acquisition through annotation confidence and quantification tier to the final effect estimate—is not yet formalized, so the calibrated uncertainty the profile promises is currently an aspiration rather than a solved problem. These limitations do not undermine the argument; rather, they define the work that a community demonstration such as the EMLB would need to complete before the profile could be considered validated rather than proposed.

## 14 Conclusion

I have argued that untargeted metabolomics-based exposomics has reached the point where its limiting factor is no longer analytical discovery but evidence integration (Hartung, 2023). The discipline is technically mature, but its synthesis is not. The gap is not bridgeable by another checklist. It requires an executable interoperability profile composed of four layers: acquisition comparability, machine-readable reporting, evidence-aware annotation, and meta-analysis-ready summary statistics, which is implemented by the existing standards working groups acting in concert, enforced by repository validators, and demonstrated by a community-scale living benchmark. This is a central challenge for the Global Exposome Forum (see text footnote 4).

The components exist. The mzML format, the mzTab-M format, the Universal Spectrum Identifier, the ISA framework, the RefMet and ChEBI nomenclatures, the GNPS/MassIVE and MetaboLights and Metabolomics Workbench repositories, the SIRIUS and CANOPUS annotation engines, the mQACC and BP4NTA quality control consortia, the MERIT and OECD regulatory frameworks, and the GA4GH human exposome data standards together provide the building blocks. What is missing is the crosswalk, the validator infrastructure, the summary-statistic schema, and the political agreement to require all four layers at deposition. None of these are technical obstacles, especially with the advent of agentic AI easing their implementation.

Three convictions underlie my argument. First, FAIR^37^ is not enough. A deposited dataset is not necessarily reusable, a reusable dataset is not necessarily meta-analyzable, and the field's habit of treating “*data shared*” as a synonym for “*data combinable*” has produced a curious situation in which exposomics has more public data than ever before but produces fewer cumulative findings per dataset than ever before. Second, unknowns should not be excluded from meta-analysis. They are first-class evidence and the very point of an untargeted approach. The Universal Spectrum Identifier and spectral similarity-based pan-repository search make it possible to treat recurring unknowns as stable, citable entities, and meta-analysis should do so. Third, annotation confidence and quantification tier should be statistical inputs to meta-analysis, not labels for the methods section. Pooling confirmed compounds with putative annotations under the pretense that they are equivalent evidence is a mistake. Stratifying by confidence and tier is straightforward, scientifically honest, and produces better-calibrated effect estimates.

The exposome is, in Wild's original framing (Wild, 2005), the counterpart of the genome. The Human Genome Project succeeded because it agreed, early and explicitly, on what counted as a deposited human reference sequence and on the formats by which the sequence would be reported. Exposomics has not yet reached its corresponding agreement. The proposal of this article is that the agreement is overdue and that the materials for it are already on the table. I have attempted, in these pages, to lay them out. The work of assembling them is, properly, the responsibility of the standards groups, the repositories, the funders, and the journals. The cost of not doing this work is large and growing, in the form of duplicated discoveries, irreproducible associations, and a steady accumulation of single-study findings that the field does not know how to combine. The cost of doing it is small and front-loaded. The remaining question is one of will.

It is worth separating what can be done immediately from what will take longer. In the near term, individual laboratories can act now, without waiting for consensus, by taking the seven concrete steps listed at the end of Section 12: depositing raw data in mzML, producing mzTab-M outputs, assigning Universal Spectrum Identifiers, reporting dual-scheme confidence with the underlying evidence, recording a quantification tier per feature, exporting structured summary statistics, and pre-registering the analytic plan. In the medium term, the standards working groups, repositories, funders, and journals must produce the three things no single laboratory can: the crosswalk among existing standards, the end-to-end four-layer validator, and the Layer 4 summary-statistics schema. In the longer term, a community-scale demonstration such as the Exposomics Meta-Analysis Living Benchmark, and ultimately a coordinated Human Exposome Project, would convert these components into a durable, cumulative resource. The immediate steps are the ones that make the later ones worthwhile.

I have also argued, in two prior articles (Sillé et al., 2020; Hartung, 2023), that the exposome is the natural successor to the genome in the post-genomic era of human health research. The argument of this third article is that the methodological infrastructure to support that succession is now within reach. It requires not new science but new consensus. The opportunity is available. The next study to be published in this field can take the seven concrete steps listed at the end of Section 12, and the cumulative effect of widespread adoption will transform the field within a decade. The whole, in this domain as in Aristotle's, can be more than the sum of its parts. But only if we agree to add it up.

## Data Availability

The original contributions presented in the study are included in the article/supplementary material, further inquiries can be directed to the corresponding author.

## Footnotes

1 GNPS (Global Natural Products Social Molecular Networking): https://gnps.ucsd.edu/ and https://ccms-ucsd.github.io/GNPSDocumentation/

2 SIRIUS (formula and structural annotation): https://bio.informatik.uni-jena.de/software/sirius/

3 NORMAN Network: https://www.norman-network.com/

4 Global Exposome Forum: https://globalexposomeforum.org

5 Exposome Moonshot Forum: https://exposomemoonshot.org

6 Metabolomics Quality Assurance and Quality Control Consortium (mQACC): https://www.mqacc.org/

7 Best Practices for Non-Targeted Analysis (BP4NTA): https://nontargetedanalysis.org/

8 MEtabolomics standaRds Initiative in Toxicology (MERIT): https://oerc.ox.ac.uk/news/merit-the-metabolomics-standards-initiative-in-toxicology/

9 HUPO Proteomics Standards Initiative (PSI), maintainers of mzML, mzTab-M, and the Universal Spectrum Identifier: https://www.psidev.info/

10 Global Alliance for Genomics and Health (GA4GH): https://www.ga4gh.org/

11 CANOPUS (compound class prediction): https://bio.informatik.uni-jena.de/software/canopus/

12
https://github.com/meowcat/MSNovelist

13 NORMAN Suspect List Exchange (NORMAN-SLE): https://www.norman-network.com/?q=suspect-list-exchange

14 Metabolomics Workbench (UCSD / NIH): https://www.metabolomicsworkbench.org/

15 MetaboLights (EMBL-EBI): https://www.ebi.ac.uk/metabolights/

16 Metabolomics Society Standards Initiative (MSI): https://metabolomicssociety.org/

17 ISA-Tab/ISA-JSON (Investigation–Study–Assay framework): https://isa-tools.org/

18 RefMet (reference nomenclature for metabolomics): https://www.metabolomicsworkbench.org/databases/refmet/ and RefMet GitHub tools: https://github.com/metabolomicsworkbench/RefMet

19
https://athleteproject.eu

20
https://www.eximious-h2020.eu

21
https://www.ephor-project.eu

22
https://www.equal-life.eu/en

23
https://expanseproject.eu

24
https://heap-exposome.eu

25
https://www.hedimed.eu/exposome-network/

26
https://longitools.org/exposome/

27
https://h2020-remedia.eu

28
https://www.hbm4eu.eu/what-we-do/exposure-and-health/

29
https://www.eu-parc.eu

30
https://github.com/BioNomad/tidyexposomics

31
https://doi.org/10.5281/zenodo.17049350

32 CASP, Critical Assessment of Structure Prediction (protein-structure prediction): https://predictioncenter.org/

33 CAMI, Critical Assessment of Metagenome Interpretation: https://cami-challenge.org/

34 CASF, Comparative Assessment of Scoring Functions (protein-ligand docking, PDBbind): https://www.pdbbind-plus.org.cn/casf; PDBbind database (host of CASF): https://www.pdbbind-plus.org.cn/

35 Pan-ReDU (pan-repository reanalysis portal across MetaboLights, Metabolomics Workbench, and GNPS/MassIVE): https://redu.gnps2.org/

36
https://www.oecd.org/en/publications/oecd-omics-reporting-framework-oorf-guidance-on-reporting-elements-for-the-regulatory-use-of-omics-data-from-laboratory-based-toxicology-studies_6bb2e6ce-en.html

37
https://www.go-fair.org/fair-principles/
