# Supplementary material for: The data substrate of exposome intelligence: an interoperability profile for untargeted metabolomics
Source: Front Artif Intell. 2026 Aug 24;9:1901969. doi: 10.3389/frai.2026.1901969 (PMC13547244; doi:10.3389/frai.2026.1901969)
Supplement: Supplementary file 1 [file Supplementary_File_1.pdf]

Appendix A — URLs of tools, standards, and resources cited

The following resources are referenced in the text. URLs are current at submission and were verified against the official source.

Standards bodies and consortia

- HUPO Proteomics Standards Initiative (PSI), maintainers of mzML, mzTab-M, and the Universal Spectrum Identifier: https://www.psidev.info/
- Metabolomics Society Standards Initiative (MSI): http://metabolomicssociety.org/
- MSI workgroups portal: https://msi-workgroups.sourceforge.net/
- MSI GitHub (Core Information for Metabolomics Reporting): https://github.com/MSI-Metabolomics-Standards-Initiative/CIMR
- Metabolomics Quality Assurance and Quality Control Consortium (mQACC): https://www.mqacc.org/
- Best Practices for Non-Targeted Analysis (BP4NTA): https://nontargetedanalysis.org/
- BP4NTA Non-Targeted Analysis Study Reporting Tool (SRT): https://nontargetedanalysis.org/srt/
- NORMAN Network: https://www.norman-network.com/
- NORMAN Suspect List Exchange (NORMAN-SLE): https://www.norman-network.com/?q=suspect-list-exchange
- NORMAN-SLE Zenodo community: https://zenodo.org/communities/norman-sle/
- MEtabolomics standaRds Initiative in Toxicology (MERIT): https://oerc.ox.ac.uk/news/merit-the-metabolomics-standards-initiative-in-toxicology/
- Global Alliance for Genomics and Health (GA4GH): https://www.ga4gh.org/
- GA4GH Human Exposome Data Standards Study Group: https://www.ga4gh.org/product/human-exposome-data-standards/
- GA4GH Phenopackets standard: https://www.ga4gh.org/product/phenopackets/

File formats and machine-readable standards

- mzML (HUPO-PSI): https://www.psidev.info/mzml
- mzTab-M (HUPO-PSI): https://hupo-psi.github.io/mzTab/
- mzTab GitHub repository: https://github.com/HUPO-PSI/mzTab
- mzTab-M development repository: https://github.com/HUPO-PSI/mzTab-M
- ISA-Tab / ISA-JSON (Investigation–Study–Assay framework): https://isa-tools.org/

Persistent identifiers and nomenclature

- Universal Spectrum Identifier (USI): https://www.psidev.info/usi
- USI GitHub specification: https://github.com/HUPO-PSI/usi
- ChEBI (Chemical Entities of Biological Interest): https://www.ebi.ac.uk/chebi/
- InChI / InChIKey (IUPAC): https://www.inchi-trust.org/

- RefMet (reference nomenclature for metabolomics): https://www.metabolomicsworkbench.org/databases/refmet/
- RefMet GitHub tools: https://github.com/metabolomicsworkbench/RefMet
- PubChem: https://pubchem.ncbi.nlm.nih.gov/
- Human Metabolome Database (HMDB): https://hmdb.ca/

Public data repositories

- MetaboLights (EMBL-EBI): https://www.ebi.ac.uk/metabolights/
- Metabolomics Workbench (UCSD / NIH): https://www.metabolomicsworkbench.org/
- GNPS (Global Natural Products Social Molecular Networking): https://gnps.ucsd.edu/
- MassIVE (Mass Spectrometry Interactive Virtual Environment): https://massive.ucsd.edu/
- Pan-ReDU (pan-repository reanalysis portal across MetaboLights, Metabolomics Workbench, and GNPS/MassIVE), the GNPS2-hosted successor to the original ReDU: https://redu.gnps2.org/
- ReDU GitHub repository: https://github.com/mwang87/ReDU-MS2-GNPS
- ReDU (metadata-curated reanalysis platform): https://redu.ucsd.edu/
- GNPS Documentation: https://ccms-ucsd.github.io/GNPSDocumentation/

Annotation, processing, and statistical tools

- SIRIUS (formula and structural annotation): https://bio.informatik.uni-jena.de/software/sirius/
- SIRIUS documentation: https://boecker-lab.github.io/docs.sirius.github.io/
- CANOPUS (compound class prediction): https://bio.informatik.uni-jena.de/software/canopus/
- CSI:FingerID (included in SIRIUS)
- DataSHIELD (federated analysis framework): https://datashield.org/
- tidyexposomics R package (Laird et al., under review) — https://github.com/BioNomad/tidyexposomics
- tidyexposomics example data (Zenodo) — https://doi.org/10.5281/zenodo.17049350

Analog community benchmarks cited as comparison

- CASP, Critical Assessment of Structure Prediction (protein-structure prediction)[1]: https://predictioncenter.org/
- CAMI, Critical Assessment of Metagenome Interpretation: https://cami-challenge.org/
- CAMI host community (Microbiome COSI): https://www.microbiome-cosi.org/cami/cami

[1] CASP funding was discontinued by NIH in 2025 and the program is now operating on interim funding from Google DeepMind.

- CASF, Comparative Assessment of Scoring Functions (protein-ligand docking, https://www.pdbbind-plus.org.cn/

- PDBbind database (host of CASF): https://www.pdbbind-plus.org.cn/casf

- ABRF Proteome Informatics Research Group (iPRG): https://www.abrf.org/proteome-informatics-iprg-

- ABRF Proteomics Research Groups (iPRG, sPRG): https://abrf.org/researchgroups/proteomics-research-group-prg/proteomeinformatics/

Pathway and ontology resources

- KEGG: https://www.kegg.jp/
- Reactome: https://reactome.org/

Regulatory and policy frameworks

- OECD Omics Reporting Framework (OORF): https://www.oecd.org/chemicalsafety/testing/oecd-omics-reporting-framework.htm
- OECD Adverse Outcome Pathway programme: https://www.oecd.org/chemicalsafety/testing/adverse-outcome-pathways-molecular-screening-and-toxicogenomics.htm
- AOP-Wiki (collaborative AOP knowledge base): https://aopwiki.org/
- Tox21 (US federal high-throughput screening collaboration): https://tox21.gov/

European Human Exposome Network and adjacent initiatives

- European Human Exposome Network (EHEN): https://www.humanexposome.eu/
- Human Biomonitoring for Europe (HBM4EU): https://www.hbm4eu.eu/
- Partnership for the Assessment of Risks from Chemicals (PARC): https://www.eu-parc.eu/
- ATHLETE project: https://athleteproject.eu/
- EXPANSE project: https://expanseproject.eu/
- EPHOR project: https://www.ephor-project.eu/
- LongITools project: https://longitools.org/

AI infrastructure, exposome consortia, and modular data ecosystems

- NIH Data Commons: https://commonfund.nih.gov/dataecosystem
- HELIX (Human Early Life Exposome): https://www.projecthelix.eu/
- NIEHS NEXUS network: https://www.nexus-exposomics.org/
- Banbury Exposomics Consortium: https://www.publichealth.columbia.edu/research/centers/center-innovative-exposomics/banbury-consortium
- BioBricks Foundation: https://biobricks.org/
